# Supplementary figures and images for: Burden of mental health problems among pregnant and postpartum women in sub-Saharan Africa: systematic review and meta-analysis protocol
Source: BMJ Open. 2023 Jun 7;13(6):e069545. doi: 10.1136/bmjopen-2022-069545 (PMC10254702; doi:10.1136/bmjopen-2022-069545)

Supplemental file 2 PRISMA flow diagram

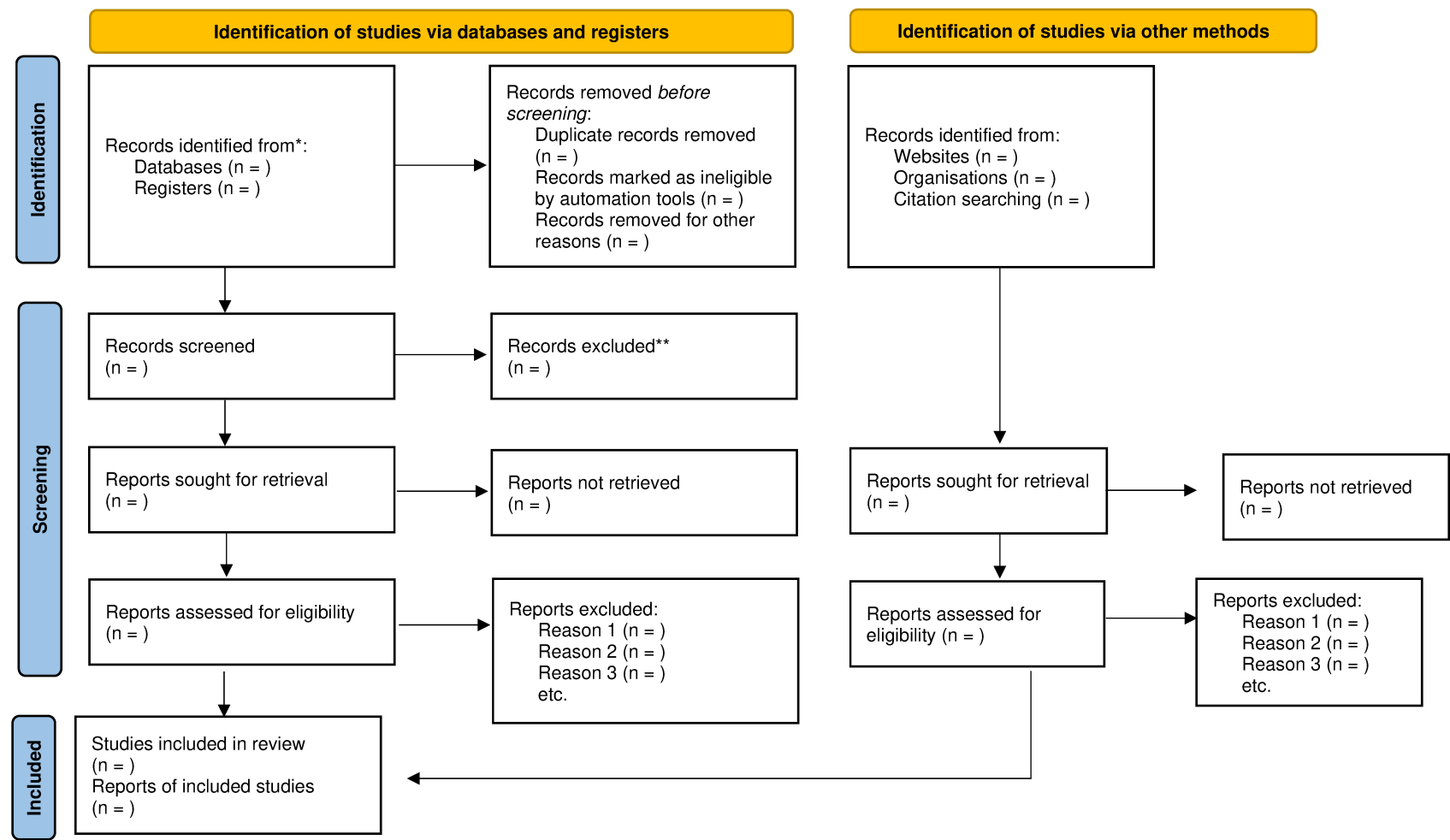

Supplement: Supplementary data [file bmjopen-2022-069545supp002.pdf]
