# Supplementary material for: Burden of mental health problems among pregnant and postpartum women in sub-Saharan Africa: systematic review and meta-analysis protocol
Source: BMJ Open. 2023 Jun 7;13(6):e069545. doi: 10.1136/bmjopen-2022-069545 (PMC10254702; doi:10.1136/bmjopen-2022-069545)
Supplement: Supplementary data [file bmjopen-2022-069545supp003.pdf]

BMJ Publishing Group Limited (BMJ) disclaims all liability and responsibility arising from any reliance placed on this supplemental material which has been supplied by the author(s)

Supplemental file 3 Data extraction sheet (adapted from Cochrane)

**1. General Information**		
1. **Date form completed** *(dd/mm/yyyy)*		
2. **Name/ID of person extracting data**		
3. **Report title** *(title of paper/ abstract/ report that data are extracted from)*		
4. **Publication type** *(e.g. full report, abstract, letter)*		
5. **Study ID** *(e.g. 01 plus surname of first author and year first full report of study was published e.g. Adjuik 2018)*		
6. **Country in which the study conducted**		
7. **Study funding source** *(including role of funders)*		
8. **Possible conflicts of interest** *(for study authors e.g. not reported)*		
**9. Study Characteristics**	**Review Inclusion Criteria** *(as defined in the Protocol)*	**Location in Text** *(page#)*
10. **Type of study**		
11. **Population description**		
12. **Focused diseases / conditions**	*Maternal mental disorders*	
13. **Types of outcome measures**	*Prevalence/incidence/risk ratios, odds ratios/mean difference/proportions*	
14. **Population description** *(from which study participants are drawn)*		
15.. **Source/setting of the population** *(e.g. urban, rural)*		
16. **Method/s of recruitment of participants**		
17. **Aim of study**		
18. **Design** *(e.g. cross-sectional study, cohort study, case-control study etc)*		
19. **Sampling technique** *(e.g. random or convenience)*		
20. **Study start date**		

21. **Study End date/duration** *(if any cohort)*		
22. **Notes:**		
23. **Total number of participants/Sample size**		
24. **Age group**		
25. **Parity**		
26. **Status of mother**	Pregnant, Post-partum	
27. **Outcomes** *(physical/observation examination: who examined?)*		
28. **Self-reported reported outcomes** *(detected by questionnaire/Tools: validated or non-validated?)*		
29. **Outcome names** *(depression, anxiety, psychosis, Birth Related Posttraumatic Stress Disorder, etc)*		
30. **Time points measured** *(report the start month/year/specify whether from start and end of intervention)*		
31. **Time points reported**		
37. **Outcome definition** *(e.g. whether standard case definition used)*		
32. **Type of measurement (***Percentage/Odds ratio/Risk ratio)*		
33. **Is outcome/tool validated?** *(Yes/No/Unclear/Not mentioned)*		
34. **Subgroup** *(if any, e.g. age-specific prevalence reporting)*		
35. **Results**		
36. **Response/non-response rate**		
37. **Any other results reported**		
38. **Unit of analysis** *(e.g. by individuals)*		
39. **Statistical methods used and appropriateness of these methods** *(e.g. proportion/%s, RR/OR)*		

40. **Whether results weighted?** ***(e.g. Yes/No)***		
41. **Any other results reported**		
42. **Unit of analysis** ***(e.g. by individuals)***		
43. **Statistical methods used and appropriateness of these methods** ***(e.g. proportion/%s, RR/OR)***		
44. **All systematic and random error adjusted?** ***(e.g. confounding, effect medication etc.)***		
45. **Strength of study**		
46. **Limitation**		
47. **Strategies to overcome the limitation**		
48. **Key conclusions of study authors**		
49. **Notes:**		
