# Supplementary material for: Burden of mental health problems among pregnant and postpartum women in sub-Saharan Africa: systematic review and meta-analysis protocol
Source: BMJ Open. 2023 Jun 7;13(6):e069545. doi: 10.1136/bmjopen-2022-069545 (PMC10254702; doi:10.1136/bmjopen-2022-069545)
Supplement: Supplementary data [file bmjopen-2022-069545supp004.pdf]

Supplemental file 4 Quality assessment checklist for prevalence studies (adapted from Hoy et al., 2012)

Name of author(s):

Year of publication:

Study title:

Risk of bias items	Risk of bias levels	Points scored
1. Was the study's target population a close representation of the national population in relation to relevant variables, e.g. age, sex, occupation?	**Yes** (**LOW RISK**): The study's target population was a close representation of the national population.	0
	**No** (**HIGH RISK**): The study's target population was clearly NOT representative of the national population.	1
2. Was the sampling frame a true or close representation of the target population?	**Yes** (**LOW RISK**): The sampling frame was a true or close representation of the target population.	0
	**No** (**HIGH RISK**): The sampling frame was NOT a true or close representation of the target population.	1
3. Was some form of random selection used to select the sample, OR, was a census undertaken?	**Yes** (**LOW RISK**): A census was undertaken, OR, some form of random selection was used to select the sample (e.g. simple random sampling, stratified random sampling, cluster sampling, systematic sampling).	0
	**No** (**HIGH RISK**): A census was NOT undertaken, AND some form of random selection was NOT used to select the sample.	1
4. Was the likelihood of non-response bias minimal?	**Yes** (**LOW RISK**): The response rate for the study was ≥75%, OR, an analysis was performed that showed no significant difference in relevant demographic characteristics between responders and non- responders	0
	**No** (**HIGH RISK**): The response rate was <75%, and if any analysis comparing responders and non-responders was done, it showed a significant difference in relevant demographic characteristics between responders and non-responders	1
5. Were data collected directly from the subjects (as opposed to a proxy)?	**Yes** (**LOW RISK**): All data were collected directly from the subjects.	0
	**No** (**HIGH RISK**): In some instances, data were collected from a proxy.	1

6.Was an acceptable case definition used in the study?	**Yes** (**LOW RISK**): An acceptable case definition was used.	0
	**No** (**HIGH RISK**): An acceptable case definition was NOT used	1
7. Was the study instrument that measured the parameter of interest (e.g. prevalence of low back pain) shown to have reliability and validity (if necessary)?	**Yes** (**LOW RISK**): The study instrument had been shown to have reliability and validity (if this was necessary), e.g. test-re- test, piloting, validation in a previous study, etc.	0
	**No** (**HIGH RISK**): The study instrument had NOT been shown to have reliability or validity (if this was necessary).	1
8. Was the same mode of data collection used for all subjects?	**Yes** (**LOW RISK**): The same mode of data collection was used for all subjects.	0
	**No** (**HIGH RISK**): The same mode of data collection was NOT used for all subjects.	1
9.Were the numerator(s) and denominator(s) for the parameter of interest appropriate	**Yes** (**LOW RISK**): The paper presented appropriate numerator(s) AND denominator(s) for the parameter of interest (e.g. the prevalence of low back pain).	0
	**No** (**HIGH RISK**): The paper did present numerator(s) AND denominator(s) for the parameter of interest but one or more of these were inappropriate.	1
10. Summary on the overall risk of study bias	**LOW RISK**	0-3
	**MODERATE RISK**	4-6
	**HIGH RISK**	7-9
