# Supplementary material for: Burden of mental health problems among pregnant and postpartum women in sub-Saharan Africa: systematic review and meta-analysis protocol
Source: BMJ Open. 2023 Jun 7;13(6):e069545. doi: 10.1136/bmjopen-2022-069545 (PMC10254702; doi:10.1136/bmjopen-2022-069545)
Supplement: Author's manuscript [file bmjopen-2022-069545.draft_revisions.pdf]

BMJ Open is committed to open peer review. As part of this commitment we make the peer review history of every article we publish publicly available.

When an article is published we post the peer reviewers' comments and the authors' responses online. We also post the versions of the paper that were used during peer review. These are the versions that the peer review comments apply to.

The versions of the paper that follow are the versions that were submitted during the peer review process. They are not the versions of record or the final published versions. They should not be cited or distributed as the published version of this manuscript.

BMJ Open is an open access journal and the full, final, typeset and author-corrected version of record of the manuscript is available on our site with no access controls, subscription charges or pay-per-view fees (http://bmjopen.bmj.com).

If you have any questions on BMJ Open's open peer review process please email info.bmjopen@bmj.com

BMJ Open

Burden of Mental Health Problems Among Pregnant and Post-Partum Women in Sub-Saharan Africa: Systematic Review Protocol

Journal:	*BMJ Open*
Manuscript ID	bmjopen-2022-069545
Article Type:	Protocol
Date Submitted by the Author:	27-Oct-2022
Complete List of Authors:	Awini, Elizabeth; Ghana Health Service<br>Agyepong, Irene; Ghana Health Service; Ghana College of Physicians and Surgeons, Faculty of Public Health<br>Owiredu, David; University of Ghana<br>Gyimah, Leveana; Pantang Hospital; Ghana College of Physicians and Surgeons, Faculty of Psychiatry<br>Ashinyo, Mary; Ghana Health Service, Institutional Care Division<br>Yevoo, Lucy; Ghana Health Service<br>Aye, Sorre Grace Emmanuelle Victoire ; Ghana Health Service, Research and Development Division; Ghana College of Physicians and Surgeons, Faculty of Public Health<br>Abbas, Shazra; The University of Melbourne, Nossal Institute for Global Health, Melbourne School of Population and Global Health<br>Cronin de Chavez, Anna; London School of Hygiene and Tropical Medicine Department of Global Health and Development<br>Kane, Sumit; The University of Melbourne, Melbourne School Of Population And Global Health<br>Mirzoev, Tolib; London School of Hygiene & Tropical Medicine, Global Health and Development<br>Danso-Appiah , Anthony ; University of Ghana, Epidemiology and Disease Control; University of Ghana, Centre for Evidence Synthesis and Policy
Keywords:	MENTAL HEALTH, Maternal medicine < OBSTETRICS, PSYCHIATRY

SCHOLARONE™ Manuscripts

Burden of Mental Health Problems Among Pregnant and Post-Partum Women in Sub-Saharan Africa: Systematic Review Protocol

Elizabeth Awini[1], Irene Akua Agyepong[1,2], David Owiredu[3], Leveana Gyimah[4,5], Mary Eyram Ashinyo[6], Linda Lucy Yevoo[1], Sorre Grace Emmanuelle Victoire Aye[1,2], Shazra Abbas[7], Anna Cronin de Chavez[8], Sumit Kane[7], Tolib Mirzoev[8], Anthony Danso-Appiah[3,9,*]

Author affiliation

[1]Dodowa Health Research Centre, Research and Development Division, Ghana Health Service, Dodowa, Ghana
[2]Ghana College of Physicians and Surgeons, Faculty of Public Health, Accra, Ghana
[3]Department of Epidemiology and Disease Control, School of Public Health, University of Ghana, Legon, Ghana
[4]Pantang Hospital, Accra, Ghana
[5]Ghana College of Physicians and Surgeons, Faculty of Psychiatry, Accra, Ghana
[6]Institutional Care Division, Ghana Health Service, Accra, Ghana
[7]Nossal Institute for Global Health, Melbourne School of Population and Global Health, The University of Melbourne, Victoria 3010 Australia
[8]Department of Global Health and Development, London School of Hygiene and Tropical Medicine, UK
[9] Centre for Evidence Synthesis and Policy, University of Ghana, Legon, Accra, Ghana

Author email addresses

Elizabeth Awini; awini.elizabeth@gmail.com
Irene Akua Agyepong; irene.agyepong@gmail.com
David Owiredu; evasupreme@gmail.com
Leveana Gyimah; leveana12@gmail.com
Mary Eyram Ashinyo; mary.ashinyo@ghsmail.org
Linda Lucy Yevoo; lyevoo@gmail.com
Sorre Grace Emmanuelle Victoire Aye; graceaye7@gmail.com
Shazra Abbas; shazra.abbas1@unimelb.edu.au
Anna Cronin de Chavez; Anna.Chavez@lshtm.ac.uk
Sumit Kane; sumit.kane@unimelb.edu.au
Tolib Mirzoev; Tolib.Mirzoev@lshtm.ac.uk

***Corresponding author**

Dr Antony Danso-Appiah, DSc, PhD

1. Department of Epidemiology and Disease Control, School of Public Health University of Ghana, P. O. Box LG13, Legon, Accra, Ghana
2. UG Centre for Evidence Synthesis and Policy, School of Public Health University of Ghana, P. O. Box LG13, Legon, Accra, Ghana

Email: adanso-appiah@ug.edu.gh
tdappiah@yahoo.co.uk

For peer review only

For peer review only - http://bmjopen.bmj.com/site/about/guidelines.xhtml

Tel: +233 269083789
+44 7958721789

Keywords:
Maternal, mental health, mental disorders, mental conditions, perinatal, pregnant women, postpartum women, burden of disease, Sub-Saharan Africa

ORCID IDs

Elizabeth Awini: **0000-0002-1147-985X**

Irene Akua Agyepong: **0000-0002.0193.5882**

David Owiredu: **0000-0002-4503-1850**

Leveana Gyimah: **0000-0003-2323-4718**

Mary Eyram Ashinyo: **0000-0002-8493-9378**

Linda Lucy Yevoo: **0000-0003-2309-2158**

Aye Sorre Grace Emmanuelle Victoire **0000-0001-7435-677X**

Shazra Abbas **0000-0002-5402-0766**

Anna Cronin de Chavez: **0000-0002-4050-4276**

Sumit Kane: **0000-0002-4858-7344**

Tolib Mirzoev: **0000-0003-2959-9187**

Anthony Danso-Appiah: **0000-0003-1747-0060**

Word count

Main Text Word Count: 2689

Tables: 1

For peer review only - http://bmjopen.bmj.com/site/about/guidelines.xhtml

Abstract

Introduction

Pregnancy and postpartum- related mental health (MMH) problems pose serious public health threat to the society, but worryingly, neglected in Sub-Saharan Africa (SSA). This review will assess the burden and distribution of MMH problems in SSA, with the aim to inform the implementation of context sensitive interventions and policies.

Methods and analysis

All relevant databases, grey literature and non-database sources will be searched. PubMed, LILAC, CINAHL, SCOPUS and PsycINFO, Google Scholar, African Index Medicus, HINARI, African Journals Online and IMSEAR will be searched from inception to 31st December 2022 without language restriction. The reference lists of articles will be reviewed, and experts contacted for additional studies missed by our searches. Study selection, data extraction and risk of bias assessment will be done independently by at least two reviewers and any discrepancies will be resolved through discussion between the reviewers. Binary outcomes will be assessed using Odds Ratio (OR) or Risk Ratio (RR), and Mean Difference (MD) for continuous outcomes; all will be presented with their 95% confidence intervals (CIs). Heterogeneity will be investigated graphically for overlapping CIs, and statistically using the I^2 statistic and where necessary subgroup analyses will be performed. Random-effects model meta-analysis will be conducted when heterogeneity is appreciable, otherwise fixed-effect model will be used. The overall level of evidence will be assessed using GRADE.

Ethics, dissemination and protocol registration

Ethical Approval is not required. Findings of this study will be disseminated through stakeholder forums, conferences and peer review publications. This review has been registered in PROSPERO-CRD42021269528.

Strengths and limitations of this study

- This review is the first to attempt to rigorously summarize the burden of MMH problems in SSA to provide country and regional- specific estimates of the burden of MMH problems with the aim to inform the implementation of context sensitive intervention and policies.

For peer review only - http://bmjopen.bmj.com/site/about/guidelines.xhtml

- Screening of articles, data extraction and quality assessment will be done by at least two independent reviewers to minimize bias and any discrepancies will be resolved through discussion between the review authors.
- Subgroup analysis will be done to explore whether burden of MMH problems varies according to setting, geographic location, age parity and others across SSA.

Introduction

Maternal mental health (MMH) problems, occurring during pregnancy and postpartum constitute a serious public health problem globally, affecting about 10% of pregnant women and 13% of postpartum mothers[1-3]. A systematic review on global depression among postpartum women estimated prevalence of 17.2% worldwide[4]. In high income countries, prevalence of postpartum depression ranged from 5% to 20%[5]. Data from 2010-2015 Northern Ireland Maternity System indicated that 18.9% of pregnant women reported a history of at least one mental disorder[6]. In the United States, 10% depression in mothers was reported over 12 months[7]. Analysis of Claims data from January to December 2008 involving 38,174 pregnant women in Germany identified at least one mental health problem from four main mental health disorders in 16,639 of the women[8] with somatoform/dissociative disorder being 24.2%, anxiety 16.9%, stress reactions 11.7%, and depression 9.3%. While reliable data are not readily available, existing estimates suggest the burden of MMH problems is relatively higher in low- and middle-income countries (LMICs) with 1 in 4 women reporting depression during pregnancy and 1 in 5 after delivery[9]. A review of evidence from LMICs on pre and postnatal depression reported prevalence of 4.9% to 50%[10] whereas a systematic review involving pre- and postnatal women living in Africa reported prevalence of depression during pregnancy at 11.3% and 18.3 % after delivery[11]. Another review in SSA reported 18.6% post-partum depression[12], with a range from 7% to 50.3%.

The common mental health problems experienced by pregnant and postpartum women are depression and anxiety[13-16]. Depression can be mild, moderate or severe. Mild depression will normally not affect the individual's ability to undertake their day-to-day activities such as self-care and interpersonal relationships whereas moderate to severe episodes can render the mother less capable of undertaking their basic self-care and that of their newborn babies which could impact on breastfeeding and bonding[17]. Other maternal mental health disorders are postpartum psychosis, pregnancy and postpartum obsessive-compulsive disorder (OCD), birth-related posttraumatic stress disorder (PTSD), intrusive thoughts and mania, schizophrenia, infanticide, substance use disorder, anorexia nervosa, bulimia, suicidal

For peer review only - http://bmjopen.bmj.com/site/about/guidelines.xhtml

ideation, bipolar affective disorder, paranoia, psychopathy, neurotic disorders and self-harm[18, 19]. These disorders may develop as a result of experiences associated with childbirth, foetal loss, congenital malformations, intimate partner violence during pregnancy, lack of partner support, history of abuse, unplanned pregnancy, complications in previous pregnancy, higher perceived stress, lower self-esteem[20-24], and many more. Individuals with predisposition to bipolar affective disorders may develop manic episodes from pregnancy-related stress[20]. Severity of maternal mental health disorders can potentially progresses from mild or moderate to severe forms if not identified early and appropriate intervention instituted[25, 26].

Rationale for this systematic review

Mothers with mental health problems may not be able to realize their abilities, work productively or cope with the normal stresses of life[3]. Maternal mental health disorders can have negative effects on both the mother and the child[27]. In severe cases, depressed mothers may have symptoms of a psychosis[4]. Affected mothers often cannot function properly, with some having suicidal tendencies[3]. Evidence shows that maternal mental health problems are associated with negative birth outcomes and may adversely affect cognitive development of the infant[28, 29], nutritional status of their infants[30], and early child wellbeing[31]. There is, however, limited knowledge to inform policy makers on the burden of maternal mental health disorder in SSA to inform on the selection and implementation of locally relevant, feasible, and effective interventions. Maternal mental health is an important health problem which should be given the needed attention but this has often been neglected as a health priority[32-34.] There is much evidence that in LMICs the vast majority of women who experience mental health problems during and after pregnancy do not receive the needed treatment[1, 35]. Although few systematic reviews have investigated mental health problems in SSA, none focused specifically on MMH[36, 37]. The only review that assessed risk factors for antenatal depression, included only studies conducted in Ethiopia[36], and although a systematic review protocol has been registered in PROSPERO[37], it intends to explore the role of MMH disorders and stillbirths.

This review will assess the burden of MMH problems among pregnant and post-partum women in SSA and attempts to provide robust country and regional estimates of the burden of disease across countries in SSA. Specifically, it will determine the prevalence and incidence and describe the socio-demographic characteristics, general obstetric histories and characteristics of women with MMH problems; assess effects of maternal mental health problems on birth outcomes; and analyze for differences in the burden

For peer review only - http://bmjopen.bmj.com/site/about/guidelines.xhtml

of mental health problems among pregnant and post-partum women between rural and urban settings. The review will answer the following specific questions: 1) What is the magnitude of MMH problems among pregnant and post-partum women living in SSA? 2) What are the socio-demographic characteristics of pregnant and post-partum women with mental health problems in SSA? 3) What are the general obstetric histories and characteristics of pregnant and post-partum women in SSA with mental health problems? 4) Is there any difference in the burden of pregnant and post-partum women with mental health problems between rural and urban settings? and 6) Are there any documented effects of birth outcomes of pregnant women with mental health problems?

Review methods

This review protocol has been prepared following the Preferred Reporting Items for Systematic Review and Meta-Analysis extension for protocols (PRISMA-P)[38] (Supplemental file 1) and the PRISMA flow diagram (Supplemental file 2). The full review will be prepared in line with the Preferred Reporting Items for Systematic Review and Meta-Analysis (PRISMA).

Criteria for considering studies for this review

Types of studies

Any study conducted in SSA that assessed burden of maternal mental health problems among pregnant and post-partum women will be eligible for inclusion. This review is not an intervention effectiveness review and randomized controlled trials (RCTs) are not the focus but if a RCT reported baseline number of cases and used a well-defined sample to serve as the denominator to allow the calculation of proportion/prevalence/incidence in pregnant or postpartum women, such an RCT will be eligible for inclusion. Reviews will not be eligible for inclusion. However, we will go through the reviews to identify potentially eligible missed by our searches. If the study is a global review having, for example, SSA or sub-regional sub-set we will extract the studies conducted in SSA for inclusion in this review. If the study reported a country or regional estimate without a well-defined sample (representative sample or sub-sample of the source population), it will not be eligible for inclusion. In cases where the results of a multi-country study have been lumped together and there is no way of disaggregating the data, such studies will not be included. Case studies and case series (these are atypical and not representative of the source population), commentaries or opinions, will not be eligible for inclusion.

For peer review only - http://bmjopen.bmj.com/site/about/guidelines.xhtml

Participants

Pregnant and post-partum (postpartum is defined as up to 12 months after delivery) women living in SSA, diagnosed of any mental health disorder (depression, anxiety, postpartum psychosis, bipolar disorders, substance misuse disorders, dysthymia, obsessive-compulsive disorder, posttraumatic stress disorder, schizophrenia, infanticide, suicidal ideation, paranoia, psychopathy, neurosis/neurotic disorders, self-harm, anorexia nervosa, bulimia, etc.) will be eligible for inclusion. The tool or criteria for diagnosis should be stated, for example, standard operational diagnostic criteria such as Research Diagnostic Criteria (RDC)[39], the 10th edition of the International Classification of Diseases (ICD-10)[40] or Diagnostic and Statistical Manual of Mental Disorders (DSM-V)[41]. Pregnant or post-partum women whose diagnosis of mental health disorder could not be confirmed will be excluded.

Interventions

This systematic review is not intervention review.

Comparison

This is non-comparative review but where outcomes or variables permit comparison, we will attempt compare

Outcomes

Primary outcomes

- Burden of MMH problems measured as prevalence, incidence etc.
- Birth outcomes (maternal and foetal outcomes) up to 12 months postpartum
- Type of MMH conditions (for diagnosed cases)

Secondary outcomes

- Proportion of MMH conditions captured in the community
- Predisposition socio-demographic characteristics of pregnant and post-partum women with mental health problems
- Predisposition obstetric histories and characteristics of pregnant and post-partum women in SSA with mental health problems
- Burden of mental health problems among pregnant and post-partum women between rural and urban settings

Search strategy

We will search the following electronic databases: PubMed, PsycINFO, LILAC and CINAHL from inception to 31st December 2022 without language restriction and using the search terms in Table 1. We will also search Google Scholar, African Index Medicus, HINARI, African Journals Online, IMSEAR and relevant preprint repositories. Grey literature including dissertations and

For peer review only - http://bmjopen.bmj.com/site/about/guidelines.xhtml

conference proceedings will be searched. Reference list of retrieved articles will be reviewed, and experts in the field of MMH will be contacted for studies not captured by our searches (see Table 1 for search strategy developed for PubMed).

Table 1 Search Strategy for PubMed (to be adapted for the other databases)

Search	Query	Results
#1	Search: **((((((((((((((((((((((((("mental health"[Title/Abstract]) OR ("mental disorder"[Title/Abstract])) OR ("mental illness"[Title/Abstract])) OR ("mental problem"[Title/Abstract])) OR ("chronic mental illness"[Title/Abstract])) OR ("psychiatric illness"[Title/Abstract])) OR ("chronic psychiatric illness"[Title/Abstract])) OR ("chronic insanity"[Title/Abstract])) OR (insanity[Title/Abstract])) OR ("chronic mental disorder"[Title/Abstract])) OR ("dementia praecox"[Title/Abstract])) OR (schizophrenia[Title/Abstract])) OR (psychoses[Title/Abstract])) OR (psychosis[Title/Abstract])) OR ("schizophrenic psychosis"[Title/Abstract])) OR (depression[Title/Abstract])) OR ("mental sickness"[Title/Abstract])) OR ("mental disease"[Title/Abstract])) OR (maladjustment[Title/Abstract])) OR ("emotional disorder"[Title/Abstract])) OR ("nervous disorder"[Title/Abstract])) OR ("nervous breakdown"[Title/Abstract])) OR (neurosis[Title/Abstract])) OR ("neurotic disorder"[Title/Abstract])) OR (psychopathy[Title/Abstract])) OR (paranoia[Title/Abstract])**	
#2	Search: **((((((((((((("pregnant women"[Title/Abstract]) OR (prepartum[Title/Abstract])) OR (peripartum[Title/Abstract])) OR (perinatal[Title/Abstract])) OR (prenatal[Title/Abstract])) OR (antenatal[Title/Abstract])) OR ("during pregnancy"[Title/Abstract])) OR (postpartum[Title/Abstract])) OR (maternal[Title/Abstract])) OR (maternity[Title/Abstract])) OR (parturient[Title/Abstract])) OR (antepartum[Title/Abstract])) OR ("post-delivery"[Title/Abstract])) OR (puerperium[Title/Abstract])**	
#3	Search: **(#1) AND (#2)**	
#4	Search: **(("sub-Saharan Africa") OR (SSA)) OR (Angola)) OR (Benin)) OR (Botswana)) OR ("Burkina Faso")) OR (Burundi)) OR (Cameroon)) OR ("Cape Verde")) OR ("Central African Republic")) OR (Chad)) OR (Comoros)) OR (Congo)) OR ("Cote d'Ivoire")) OR (Djibouti)) OR ("Equatorial Guinea")) OR (Ethiopia)) OR (Gabon)) OR ("The Gambia")) OR (Ghana)) OR (Guinea)) OR ("Guinea-Bissau")) OR (Kenya)) OR (Lesotho)) OR (Liberia)) OR (Madagascar)) OR (Malawi)) OR (Mali)) OR (Mauritania)) OR (Mauritius)) OR (Mozambique)) OR (Namibia)) OR (Niger)) OR (Nigeria)) OR (Rwanda)) OR ("Sao Tome and Principe")) OR (Senegal)) OR (Seychelles)) OR ("Sierra Leone")) OR (Somalia)) OR ("South Africa")) OR (Sudan)) OR (Swaziland)) OR (Tanzania)) OR (Togo)) OR (Uganda)) OR (Zaire)) OR (Zambia)) OR (Zimbabwe)**	
#5	Search: **(#3) AND (#4)**	

For peer review only - http://bmjopen.bmj.com/site/about/guidelines.xhtml

The search strategy will be adapted as appropriate for other databases. All searches will be re-run just before the final analyses and any further eligible studies identified will be included.

Study selection

The search output will be managed, collated and de-duplicated using EndNote. The deduplicated articles will be exported to Rayyan[42] for screening and selection. At least two reviewers will screen titles and abstracts of identified studies independently using pre-specified and piloted eligibility flowchart (Figure 1). The full text of potentially relevant studies will also be reviewed independently for inclusion. The PRISMA flow diagram will be used to document the flow of studies and reasons for exclusion. Discrepancies will be resolved through discussion between the reviewers.

Data extraction and management

A validated data extraction sheet adapted from the Cochrane Collaboration (Supplemental file 3) will be used by the reviewers to independently extract relevant data. Data to be extracted include characteristics of the studies such as year study was conducted, year study was published (for published studies), country where study was conducted, study design and sample size; socio-demographic characteristics of the participants (age, setting, socio-economic status, level of education and occupation); obstetric factors (parity, maternal age, age at first delivery, history of miscarriage, history of stillbirth), mental health conditions (depression, anxiety, postpartum psychosis, dysthymia/persistent depressive disorder, pregnancy and postpartum obsessive-compulsive disorder (OCD), birth- related posttraumatic stress disorder (PTSD), intrusive thoughts and mania, schizophrenia, infanticide, substance use disorder, anorexia nervosa, bulimia, suicidal ideation, bipolar affective disorder, paranoia, psychopathy, neurotic disorders and self-harm); and quality domains for the assessment of quality of the included studies for risk of bias. Data on burden of the disease (such as prevalence, incidence and duration of the MMH problem) will be extracted. The corresponding authors of the primary studies will be contacted for missing data or unclear information. Where it is not possible to obtain the missing information, data will be analyzed based on those with complete outcome data and the amount of data missing with reasons will be provided. If necessary, data will be coded and recoded before use in the analysis. The extracted data will be verified independently and any disagreement will be resolved through discussion.

For peer review only - http://bmjopen.bmj.com/site/about/guidelines.xhtml

Assessment of quality of the included studies

At least two reviewers will assess quality of the included studies for risk of bias (methodology and reporting) independently using the appropriate risk of bias assessment tools. Since this review is not focusing on randomized controlled trials (RCTs), the domains on the Cochrane Risk of Bias (ROB) tool[43] will not be covered fully. Selective outcome and analysis reporting bias (which occurs when studies with positive and significant results are likely to be reported or published) are the domains to be considered on the Cochrane ROB tool. The Cochrane criteria for reporting risk of bias in the included studies will be used to assess pre-specified outcomes of interest. The risk of bias will be rated as "low" if protocol of the study is available and all pre-specified outcomes of interest as specified in the protocol, or the protocol is not available but it is clear that all pre-specified and expected outcomes of interest have been reported. The risk of bias will be rated as "high" for a study if outcomes are not reported as pre-specified or expected and "unclear" when there is not enough information to make clear judgement. Other pre-specified biases pertaining to the methods, source of funding etc will be assessed and rated. The Risk of Bias Tool for Prevalence studies will be assessed using the tool by Hoy et al[44] (Supplemental file 4) on four domains: selection bias, non-response bias, measurement bias and bias related to data analysis for prevalence studies. Each risk of bias domain will be graded as 'low risk', 'high risk', and 'unclear' risk of bias. Any disagreements will be resolved through discussion between the reviewers, and if necessary, a third reviewer will be consulted.

Data analysis

Binary outcomes will be assessed using Odds Ratio (OR) or Risk Ratio (RR), and for continuous data, we will use Mean difference (MD) or Standardized Mean Difference (SMD) for means that used different scales. Meta-Analysis, the statistical component of systematic review, will be used to combine study outcomes. Odds Ratio, RR and MD of the individual studies will be pooled and presented with their 95% confidence interval (CI). Random-effects model will be used in the meta-analysis when heterogeneity is high, otherwise fixed-effect model will be used. Descriptive statistics will be used to describe proportions (prevalence and incidence).

Heterogeneity and subgroup analysis

Heterogeneity arises because of variation in the study design, characteristics of participants or outcomes between or within studies[45]. Heterogeneity will be investigated both graphically and statistically. The I^2 statistic which describes the percentage of variability that is due to heterogeneity rather than chance will be estimated. The I^2 is classified into four levels: 0%, 1-29%,

For peer review only - http://bmjopen.bmj.com/site/about/guidelines.xhtml

30-59% and 60-100%[46] and $I^2>50\%$ indicates significant heterogeneity[47]. Subgroup analysis which is used to estimate an effect of indicator within each subgroup or subset will be used to address heterogeneity due to variation in effects in each subgroup mixed effect model. Thus, the burden of MMH problems will be estimated separated for urban and rural and for each age group. Subgroup analysis will also be done for gestational age and postpartum period. If we find sufficient number of studies, subgroup analysis will also be based on perinatal mental disorder type, study design, parity and location.

Grading the evidence

The overall evidence of the systematic review will be graded using Grading of Recommendations Assessment, Development and Evaluation (GRADE)[48] (Available from guidelinedevelopment.org/handbook). The GRADE system assesses the following domains: risk of bias, imprecision, inconsistency, indirectness and publication bias, and classifies the quality of evidence as high, moderate, low, and very low. If evidence from a study is graded high quality, it implies further research is very unlikely to change the confidence in the estimate of effect while a grading of very low quality implies an estimate of effect is very doubtful. This protocol has been registered on the International Prospective Register of Systematic Reviews (PROSPERO) with registration **ID CRD42021269528.**

Discussion

It is anticipated that this systematic review will contribute critical evidence on estimates of the burden of maternal mental health problems and birth outcomes of mothers with mental health problems in Sub-Saharan Africa. To the best of our knowledge, this is the first review to summarise the burden of MMH problems in SSA and provide country-specific and regional estimates. Subgroup analysis will be done to find out whether the burden of MMH problems vary by residence, age, gestation age of postpartum period. It will also describe socio-demographic characteristics, general obstetric histories and characteristics of women with MMH problems, analyze for differences in the burden of mental health problems among pregnant and post-partum women between rural and urban settings. This systematic review forms a major component of the RESPONSE Project investigating Health System Responsiveness to neglected Health Needs of Vulnerable Groups in Ghana and Vietnam (https://www.lshtm.ac.uk/research/centres-projects-groups/response).

For peer review only - http://bmjopen.bmj.com/site/about/guidelines.xhtml

Patient and public involvement

The review questions and outcome measures have been developed collaboratively with the relevant patient and consumer involment and informed by their priorities, experience, and preferences in line with GRIPP2 reporting checklists. The review findings will be shared with relevant wider patient communities who will also be involved in the results dissemination.

Ethical approval and dissemination

Ethical Approval is not required to conduct this review**.** The results of the review will be presented to stakeholders (policymakers and practitioners), It will also be disseminated through conferences and peer review publications.

Author contributions

Protocol conceptualization and design: EA, IAA, SK, TM and ADA

Drafting the work or revising it critically for important intellectual content: All authors

Acquisition, analysis, or interpretation of data: All authors

Agreement to be accountable for accuracy or integrity of all aspects of the work: All authors

Final approval of the version to be published: All authors

Supervision of this work: ADA

Acknowledgments

This systematic review was supported by the capacity building initiative of the UG Centre for Evidence Synthesis and Policy (UGCESP), Africa Communities of Evidence Synthesis and Translation (ACEST) and the RESPONSE Project that are jointly training experts in evidence synthesis and translation across Low and Middle-Income Countries (LMICs).

Funding Statement

This review is part of a wider RESPONSE study, which received funding from the Joint MRC/ESRC/DFID/Wellcome Health Systems Research Initiative (grant ref: MR/T023481/1). The views expressed in this publication are those of the author(s) and not necessarily those of the funders.

Competing of interest: None declared.

For peer review only

For peer review only - http://bmjopen.bmj.com/site/about/guidelines.xhtml

References

1. Kathree T, Selohilwe OM, Bhana A, Petersen I. Perceptions of postnatal depression and health care needs in a South African sample: the "mental" in maternal health care. *BMC Women's Health.* 2014;14(1):1-11.

2. McCauley M, Brown A, Ofosu B, Van Den Broek N. "I just wish it becomes part of routine care": healthcare providers' knowledge, attitudes and perceptions of screening for maternal mental health during and after pregnancy: a qualitative study. *BMC psychiatry.* 2019;19(1):1-8.

3. WHO. WHO | Maternal mental health. Available at: https://www.who.int/mental_health/maternal-child/maternal_mental_health/en/. Accessed 20 December 2020, 2020.

4. Wang Z, Liu J, Shuai H, et al. Mapping global prevalence of depression among postpartum women. *Translational psychiatry.* 2021;11(1):1-13.

5. Nilaweera I, Doran F, Fisher J. Prevalence, nature and determinants of postpartum mental health problems among women who have migrated from South Asian to high-income countries: a systematic review of the evidence. *Journal of Affective Disorders.* 2014;166:213-226.

6. Mongan D, Lynch J, Hanna D, et al. Prevalence of self-reported mental disorders in pregnancy and associations with adverse neonatal outcomes: a population-based cross-sectional study. *BMC pregnancy and childbirth.* 2019;19(1):1-10.

7. Ertel KA, Rich-Edwards JW, Koenen KC. Maternal depression in the United States: Nationally representative rates and risks. *Journal of Women's Health.* 2011;20(11):1609-1617.

8. Wallwiener S, Goetz M, Lanfer A, et al. Epidemiology of mental disorders during pregnancy and link to birth outcome: a large-scale retrospective observational database study including 38,000 pregnancies. *Archives of gynecology and obstetrics.* 2019;299(3):755-763.

9. Gelaye B, Rondon MB, Araya R, Williams MA. Epidemiology of maternal depression, risk factors, and child outcomes in low-income and middle-income countries. *The Lancet Psychiatry.* 2016;3(10):973-982.

10. Parsons CE, Young KS, Rochat TJ, Kringelbach ML, Stein A. Postnatal depression and its effects on child development: a review of evidence from low-and middle-income countries. *British medical bulletin.* 2012;101(1):57-79.

11. Sawyer A, Ayers S, Smith H. Pre-and postnatal psychological wellbeing in Africa: a systematic review. *Journal of affective disorders.* 2010;123(1-3):17-29.

12. Woldeyohannes D, Tekalegn Y, Sahiledengle B, Ermias D, Ejajo T, Mwanri L. Effect of postpartum depression on exclusive breast-feeding practices in sub-Saharan Africa countries: a systematic review and meta-analysis. *BMC Pregnancy and Childbirth.* 2021;21(1):1-10.

13. Atif N, Lovell K, Rahman A. Maternal mental health: The missing "m" in the global maternal and child health agenda. Paper presented at: Seminars in perinatology, 2015.

14. Banti S, Mauri M, Oppo A, et al. From the third month of pregnancy to 1 year postpartum. Prevalence, incidence, recurrence, and new onset of depression. Results from the Perinatal Depression–Research & Screening Unit study. *Comprehensive psychiatry.* 2011;52(4):343-351.

15. Garthus-Niegel S, von Soest T, Knoph C, Simonsen TB, Torgersen L, Eberhard-Gran M. The influence of women's preferences and actual mode of delivery on post-traumatic stress symptoms following childbirth: a population-based, longitudinal study. *BMC pregnancy and childbirth.* 2014;14(1):1-10.

16. Falah-Hassani K, Shiri R, Dennis C-L. Prevalence and risk factors for comorbid postpartum depressive symptomatology and anxiety. *Journal of affective disorders.* 2016;198:142-147.

For peer review only - http://bmjopen.bmj.com/site/about/guidelines.xhtml

17. Park LT, Zarate Jr CA. Depression in the primary care setting. *New England Journal of Medicine.* 2019;380(6):559-568.

18. Bauer A, Parsonage M, Knapp M, Iemmi V, Adelaja B, Hogg S. The costs of perinatal mental health problems. *London: Centre for Mental Health and London School of Economics.* 2014.

19. Collardeau F, Corbyn B, Abramowitz J, Janssen PA, Woody S, Fairbrother N. Maternal unwanted and intrusive thoughts of infant-related harm, obsessive-compulsive disorder and depression in the perinatal period: study protocol. *BMC psychiatry.* 2019;19(1):1-15.

20. Biaggi A, Conroy S, Pawlby S, Pariante CM. Identifying the women at risk of antenatal anxiety and depression: a systematic review. *Journal of affective disorders.* 2016;191:62-77.

21. Silva MMdJ, Nogueira DA, Clapis MJ, Leite EPRC. Anxiety in pregnancy: prevalence and associated factors. *Revista da Escola de Enfermagem da USP.* 2017;51.

22. Ogbo FA, Eastwood J, Hendry A, et al. Determinants of antenatal depression and postnatal depression in Australia. *BMC psychiatry.* 2018;18(1):1-11.

23. Milgrom J, Hirshler Y, Reece J, Holt C, Gemmill AW. Social support—a protective factor for depressed perinatal women? *International journal of environmental research and public health.* 2019;16(8):1426.

24. Koh M, Ahn S, Kim J, Park S, Oh J. Pregnant women's antenatal depression and influencing factors. *Korean Journal of Women Health Nursing.* 2019;25(1):112-123.

25. Sadock BSV. Postpartum Psychiatric Syndromes. *Comprehensive Textbook of Psychiatry.* Vol 1. 7 ed: Lippincott & Wilkins Publishers.; 2000:992–1053.

26. Semple D, & Smyth, R. Disorders related to childbirth. *Oxford Handbook of Psychiatry*. 3rd ed. Oxford: Oxford University Press; 2013:470–472.

27. O'hara MW, McCabe JE. Postpartum depression: current status and future directions. *Annu Rev Clin Psychol.* 2013;9(1):379-407.

28. Tran TD, Biggs B-A, Tran T, et al. Impact on infants' cognitive development of antenatal exposure to iron deficiency disorder and common mental disorders. *Plos one.* 2013;8(9):e74876.

29. Paschetta E, Berrisford G, Coccia F, et al. Perinatal psychiatric disorders: an overview. *American Journal of Obstetrics and Gynecology.* 2014;210(6):501-509. e506.

30. Hassan BK, Werneck GL, Hasselmann MH. Maternal mental health and nutritional status of six-month-old infants. *Revista de saude publica.* 2016;50:7.

31. MacGinty RP, Lesosky M, Barnett W, Stein DJ, Zar HJ. Associations between maternal mental health and early child wheezing in a South African birth cohort. *Pediatric pulmonology.* 2018;53(6):741-754.

32. O'Mahony JM, Donnelly TT. How does gender influence immigrant and refugee women's postpartum depression help-seeking experiences? *Journal of psychiatric and mental health nursing.* 2013;20(8):714-725.

33. Bagadia A, Chandra PS. Starting the conversation-Integrating mental health into maternal health care in India. *The Indian journal of medical research.* 2017;145(3):267.

34. Cheung FK, Snowden LR. Community mental health and ethnic minority populations. *Community Mental Health Journal.* 1990;26(3):277-291.

35. Fonseca A, Canavarro MC. Women's intentions of informal and formal help-seeking for mental health problems during the perinatal period: The role of perceived encouragement from the partner. *Midwifery.* 2017;50:78-85.

36. Getinet W, Amare T, Boru B, Shumet S, Worku W, Azale T. Prevalence and risk factors for antenatal depression in Ethiopia: systematic review. *Depression research and treatment.* 2018;2018.

For peer review only - http://bmjopen.bmj.com/site/about/guidelines.xhtml

37. Adane AA, Bailey HD, Marriott R, et al. Role of maternal mental health disorders on stillbirth and infant mortality risk: a protocol for a systematic review and meta-analysis. *BMJ open.* 2020;10(5):e036280.

38. Moher D, Shamseer L, Clarke M, et al. Preferred reporting items for systematic review and meta-analysis protocols (PRISMA-P) 2015 statement. *Systematic reviews.* 2015;4(1):1-9.

39. Spitzer RL EJ, Robins E. Research Diagnostic Criteria: Rationale and Reliability. *Arch Gen Psychiatry.* 1978;35(6):773–782.

40. WHO. The ICD-10 Classification of Mental and Behavioural Disorders. *Clinical description and diagnostic guidelines*; 1993.

41. Nuckols CC, Nuckols CC. The diagnostic and statistical manual of mental disorders, (DSM-5). *Philadelphia: American Psychiatric Association.* 2013.

42. Harrison H, Griffin SJ, Kuhn I, Usher-Smith JA. Software tools to support title and abstract screening for systematic reviews in healthcare: an evaluation. *BMC medical research methodology.* 2020;20(1):1-12.

43. Higgins JP, Altman DG, Gøtzsche PC, et al. The Cochrane Collaboration's tool for assessing risk of bias in randomised trials. *Bmj.* 2011;343.

44. Hoy D, Brooks P, Woolf A, et al. Assessing risk of bias in prevalence studies: modification of an existing tool and evidence of interrater agreement. *Journal of clinical epidemiology.* 2012;65(9):934-939.

45. Spineli LM, Pandis N. Exploring heterogeneity in meta-analysis: Subgroup analysis. Part 1. *American journal of orthodontics and dentofacial orthopedics.* 2020;158(2):302-304. e301.

46. Koletsi D, Fleming PS, Michelaki I, Pandis N. Heterogeneity in Cochrane and non-Cochrane meta-analyses in orthodontics. *Journal of dentistry.* 2018;74:90-94.

47. Chang TH, Chou CC, Chang LY. Effect of obesity and body mass index on coronavirus disease 2019 severity: a systematic review and meta-analysis. *Obesity Reviews.* 2020;21(11):e13089.

48. Schünemann H. *The GRADE handbook*: Cochrane Collaboration; 2013.

For peer review only

For peer review only - http://bmjopen.bmj.com/site/about/guidelines.xhtml

Figure 1 Study selection flowchart

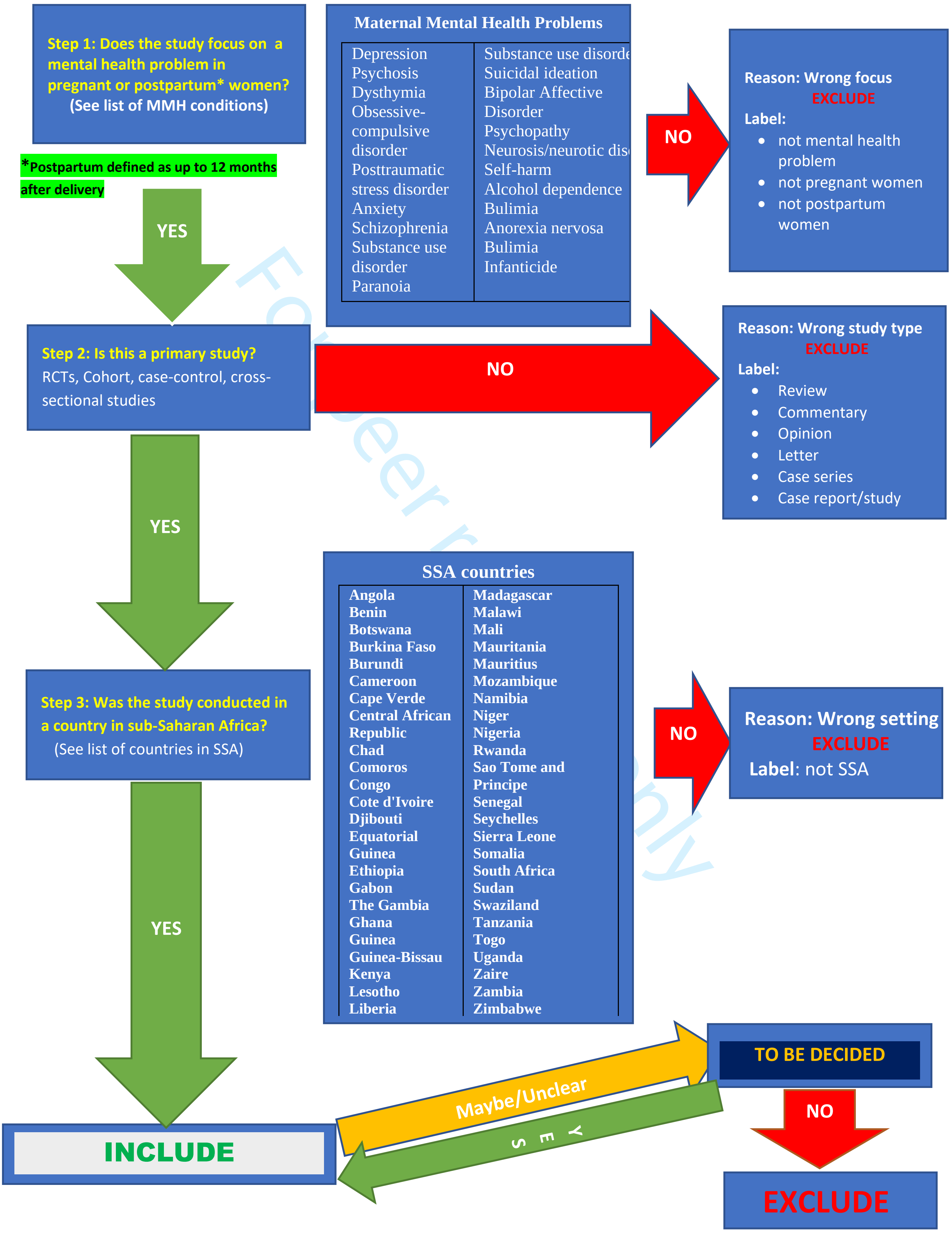

For peer review only

Supplemental file 1 Preferred Reporting Items for Systematic review and Meta-Analysis Protocols (PRISMA-P) checklist*

Section and topic	Item No	Checklist item
**ADMINISTRATIVE INFORMATION**		
Title:		
Identification	1a	Identify the report as a protocol of a systematic review
Update	1b	If the protocol is for an update of a previous systematic review, identify as such
Registration	2	If registered, provide the name of the registry (such as PROSPERO) and registration number
Authors:		
Contact	3a	Provide name, institutional affiliation, e-mail address of all protocol authors; provide physical mailing address of corresponding author
Contributions	3b	Describe contributions of protocol authors and identify the guarantor of the review
Amendments	4	If the protocol represents an amendment of a previously completed or published protocol, identify as such and list changes; otherwise, state plan for documenting important protocol amendments
Support:		
Sources	5a	Indicate sources of financial or other support for the review
Sponsor	5b	Provide name for the review funder and/or sponsor
Role of sponsor or funder	5c	Describe roles of funder(s), sponsor(s), and/or institution(s), if any, in developing the protocol
**INTRODUCTION**		
Rationale	6	Describe the rationale for the review in the context of what is already known
Objectives	7	Provide an explicit statement of the question(s) the review will address with reference to participants, interventions, comparators, and outcomes (PICO)
**METHODS**		
Eligibility criteria	8	Specify the study characteristics (such as PICO, study design, setting, time frame) and report characteristics (such as years considered, language, publication status) to be used as criteria for eligibility for the review
Information sources	9	Describe all intended information sources (such as electronic databases, contact with study authors, trial registers or other grey literature sources) with planned dates of coverage
Search strategy	10	Present draft of search strategy to be used for at least one electronic database, including planned limits, such that it could be repeated
Study records:		
Data management	11a	Describe the mechanism(s) that will be used to manage records and data throughout the review
Selection process	11b	State the process that will be used for selecting studies (such as two independent reviewers) through each phase of the review (that is, screening, eligibility and inclusion in meta-analysis)
Data collection process	11c	Describe planned method of extracting data from reports (such as piloting forms, done independently, in duplicate), any processes for obtaining and confirming data from investigators
Data items	12	List and define all variables for which data will be sought (such as PICO items, funding sources), any pre-planned data assumptions and simplifications
Outcomes and prioritization	13	List and define all outcomes for which data will be sought, including prioritization of main and additional outcomes, with rationale

For peer review only

Risk of bias in individual studies	14	Describe anticipated methods for assessing risk of bias of individual studies, including whether this will be done at the outcome or study level, or both; state how this information will be used in data synthesis
Data synthesis	15a	Describe criteria under which study data will be quantitatively synthesized
	15b	If data are appropriate for quantitative synthesis, describe planned summary measures, methods of handling data and methods of combining data from studies, including any planned exploration of consistency (such as I^2, Kendall's τ)
	15c	Describe any proposed additional analyses (such as sensitivity or subgroup analyses, meta-regression)
	15d	If quantitative synthesis is not appropriate, describe the type of summary planned
Meta-bias(es)	16	Specify any planned assessment of meta-bias(es) (such as publication bias across studies, selective reporting within studies)
Confidence in cumulative evidence	17	Describe how the strength of the body of evidence will be assessed (such as GRADE)

* It is strongly recommended that this checklist be read in conjunction with the PRISMA-P Explanation and Elaboration (cite when available) for important clarification on the items. Amendments to a review protocol should be tracked and dated. The copyright for PRISMA-P (including checklist) is held by the PRISMA-P Group and is distributed under a Creative Commons Attribution Licence 4.0.

For peer review only

Supplemental file 2 PRISMA flow diagram

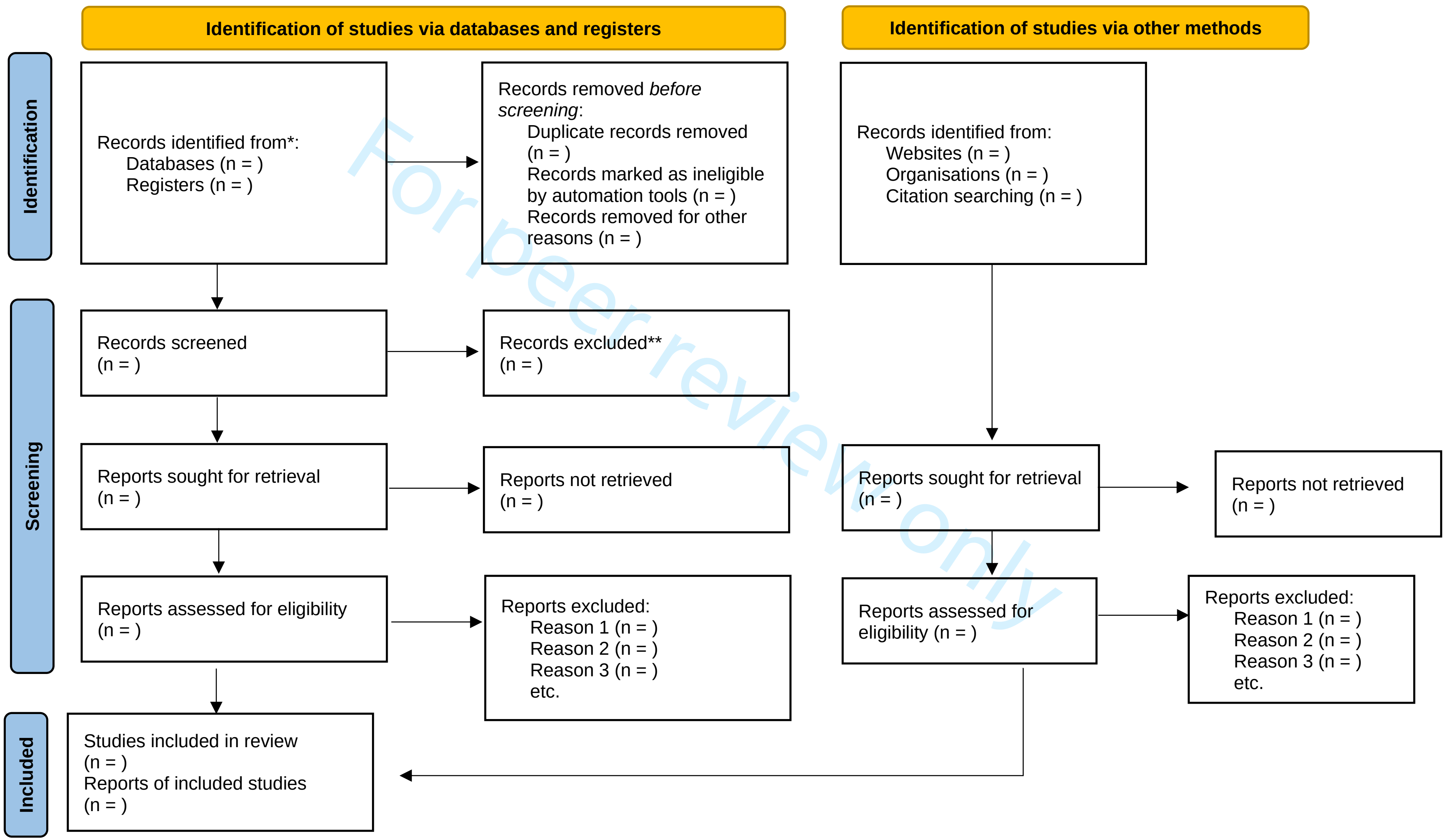

For peer review only

Supplemental file 3 Data extraction sheet (adapted from Cochrane)

**1. General Information**		
1. **Date form completed** *(dd/mm/yyyy)*		
2. **Name/ID of person extracting data**		
3. **Report title** *(title of paper/ abstract/ report that data are extracted from)*		
4. **Publication type** *(e.g. full report, abstract, letter)*		
5. **Study ID** *(e.g. 01 plus surname of first author and year first full report of study was published e.g. Adjuik 2018)*		
6. **Country in which the study conducted**		
7. **Study funding source** *(including role of funders)*		
8. **Possible conflicts of interest** *(for study authors e.g. not reported)*		
**9. Study Characteristics**	**Review Inclusion Criteria** *(as defined in the Protocol)*	**Location in Text** *(page#)*
10. **Type of study**		
11. **Population description**		
12. **Focused diseases / conditions**	*Maternal mental disorders*	
13. **Types of outcome measures**	*Prevalence/incidence/risk ratios, odds ratios/mean difference/proportions*	
14. **Population description** *(from which study participants are drawn)*		
15.. **Source/setting of the population** *(e.g. urban, rural)*		
16. **Method/s of recruitment of participants**		
17. **Aim of study**		
18. **Design** *(e.g. cross-sectional study, cohort study, case-control study etc)*		
19. **Sampling technique** *(e.g. random or convenience)*		
20. **Study start date**		

For peer review only

21. **Study End date/duration** *(if any cohort)*		
22. **Notes:**		
23. **Total number of participants/Sample size**		
24. **Age group**		
25. **Parity**		
26. **Status of mother**	Pregnant, Post-partum	
27. **Outcomes** *(physical/observation examination: who examined?)*		
28. **Self-reported reported outcomes** *(detected by questionnaire/Tools: validated or non-validated?)*		
29. **Outcome names** *(depression, anxiety, psychosis, Birth Related Posttraumatic Stress Disorder, etc)*		
30. **Time points measured** *(report the start month/year/specify whether from start and end of intervention)*		
31. **Time points reported**		
37. **Outcome definition** *(e.g. whether standard case definition used)*		
32. **Type of measurement (***Percentage/Odds ratio/Risk ratio)*		
33. **Is outcome/tool validated?** *(Yes/No/Unclear/Not mentioned)*		
34. **Subgroup** *(if any, e.g. age-specific prevalence reporting)*		
35. **Results**		
36. **Response/non-response rate**		
37. **Any other results reported**		
38. **Unit of analysis** *(e.g. by individuals)*		
39. **Statistical methods used and appropriateness of these methods** *(e.g. proportion/%s, RR/OR)*		

For peer review only

40. **Whether results weighted?** *(e.g. Yes/No)*		
41. **Any other results reported**		
42. **Unit of analysis** *(e.g. by individuals)*		
43. **Statistical methods used and appropriateness of these methods** *(e.g. proportion/%s, RR/OR)*		
44. **All systematic and random error adjusted?** *(e.g. confounding, effect medication etc.)*		
45. **Strength of study**		
46. **Limitation**		
47. **Strategies to overcome the limitation**		
48. **Key conclusions of study authors**		
49. **Notes:**		

For peer review only

Supplemental file 4 Quality assessment checklist for prevalence studies (adapted from Hoy et al., 2012)

Name of author(s):

Year of publication:

Study title:

Risk of bias items	Risk of bias levels	Points scored
1. Was the study's target population a close representation of the national population in relation to relevant variables, e.g. age, sex, occupation?	**Yes** (**LOW RISK**): The study's target population was a close representation of the national population.	0
	**No** (**HIGH RISK**): The study's target population was clearly NOT representative of the national population.	1
2. Was the sampling frame a true or close representation of the target population?	**Yes** (**LOW RISK**): The sampling frame was a true or close representation of the target population.	0
	**No** (**HIGH RISK**): The sampling frame was NOT a true or close representation of the target population.	1
3. Was some form of random selection used to select the sample, OR, was a census undertaken?	**Yes** (**LOW RISK**): A census was undertaken, OR, some form of random selection was used to select the sample (e.g. simple random sampling, stratified random sampling, cluster sampling, systematic sampling).	0
	**No** (**HIGH RISK**): A census was NOT undertaken, AND some form of random selection was NOT used to select the sample.	1
4. Was the likelihood of non-response bias minimal?	**Yes** (**LOW RISK**): The response rate for the study was ≥75%, OR, an analysis was performed that showed no significant difference in relevant demographic characteristics between responders and non- responders	0
	**No** (**HIGH RISK**): The response rate was <75%, and if any analysis comparing responders and non-responders was done, it showed a significant difference in relevant demographic characteristics between responders and non-responders	1
5. Were data collected directly from the subjects (as opposed to a proxy)?	**Yes** (**LOW RISK**): All data were collected directly from the subjects.	0
	**No** (**HIGH RISK**): In some instances, data were collected from a proxy.	1

For peer review only

6.Was an acceptable case definition used in the study?	**Yes (LOW RISK)**: An acceptable case definition was used.	0
	**No (HIGH RISK)**: An acceptable case definition was NOT used	1
7. Was the study instrument that measured the parameter of interest (e.g. prevalence of low back pain) shown to have reliability and validity (if necessary)?	**Yes (LOW RISK)**: The study instrument had been shown to have reliability and validity (if this was necessary), e.g. test-re- test, piloting, validation in a previous study, etc.	0
	**No (HIGH RISK)**: The study instrument had NOT been shown to have reliability or validity (if this was necessary).	1
8. Was the same mode of data collection used for all subjects?	**Yes (LOW RISK)**: The same mode of data collection was used for all subjects.	0
	**No (HIGH RISK)**: The same mode of data collection was NOT used for all subjects.	1
9.Were the numerator(s) and denominator(s) for the parameter of interest appropriate	**Yes (LOW RISK)**: The paper presented appropriate numerator(s) AND denominator(s) for the parameter of interest (e.g. the prevalence of low back pain).	0
	**No (HIGH RISK)**: The paper did present numerator(s) AND denominator(s) for the parameter of interest but one or more of these were inappropriate.	1
10. Summary on the overall risk of study bias	**LOW RISK**	0-3
	**MODERATE RISK**	4-6
	**HIGH RISK**	7-9

For peer review only

Reporting checklist for protocol of a systematic review and meta-analysis.

Based on the PRISMA-P guidelines.

Instructions to authors

Complete this checklist by entering the page numbers from your manuscript where readers will find each of the items listed below.

Your article may not currently address all the items on the checklist. Please modify your text to include the missing information. If you are certain that an item does not apply, please write "n/a" and provide a short explanation.

Upload your completed checklist as an extra file when you submit to a journal.

In your methods section, say that you used the PRISMA-Preporting guidelines, and cite them as:

Moher D, Shamseer L, Clarke M, Ghersi D, Liberati A, Petticrew M, Shekelle P, Stewart LA. Preferred Reporting Items for Systematic Review and Meta-Analysis Protocols (PRISMA-P) 2015 statement. Syst Rev. 2015;4(1):1.

		Reporting Item	Page Number
**Title**			
Identification	#1a	Identify the report as a protocol of a systematic review	1
Update	#1b	If the protocol is for an update of a previous systematic review, identify as such	n/a
**Registration**			
	#2	If registered, provide the name of the registry (such as PROSPERO) and registration number	PROSPERO CRD42021269528
**Authors**			
Contact	#3a	Provide name, institutional affiliation, e-mail address of all protocol authors; provide physical mailing address of corresponding author	1, 2

For peer review only

Contribution	#3b	Describe contributions of protocol authors and identify the guarantor of the review	12
**Amendments**			
	#4	If the protocol represents an amendment of a previously completed or published protocol, identify as such and list changes; otherwise, state plan for documenting important protocol amendments	n/a
**Support**			
Sources	#5a	Indicate sources of financial or other support for the review	12
Sponsor	#5b	Provide name for the review funder and / or sponsor	12
Role of sponsor or funder	#5c	Describe roles of funder(s), sponsor(s), and / or institution(s), if any, in developing the protocol	12
**Introduction**			
Rationale	#6	Describe the rationale for the review in the context of what is already known	5
Objectives	#7	Provide an explicit statement of the question(s) the review will address with reference to participants, interventions, comparators, and outcomes (PICO)	5, 6
**Methods**			
Eligibility criteria	#8	Specify the study characteristics (such as PICO, study design, setting, time frame) and report characteristics (such as years considered, language, publication status) to be used as criteria for eligibility for the review	6, 7
Information sources	#9	Describe all intended information sources (such as electronic databases, contact with study authors, trial registers or other grey literature sources) with planned dates of coverage	7, 8

For peer review only

Search strategy	#10	Present draft of search strategy to be used for at least one electronic database, including planned limits, such that it could be repeated	8
Study records - data management	#11a	Describe the mechanism(s) that will be used to manage records and data throughout the review	9
Study records - selection process	#11b	State the process that will be used for selecting studies (such as two independent reviewers) through each phase of the review (that is, screening, eligibility and inclusion in meta-analysis)	9
Study records - data collection process	#11c	Describe planned method of extracting data from reports (such as piloting forms, done independently, in duplicate), any processes for obtaining and confirming data from investigators	9
Data items	#12	List and define all variables for which data will be sought (such as PICO items, funding sources), any pre-planned data assumptions and simplifications	9
Outcomes and prioritization	#13	List and define all outcomes for which data will be sought, including prioritization of main and additional outcomes, with rationale	7
Risk of bias in individual studies	#14	Describe anticipated methods for assessing risk of bias of individual studies, including whether this will be done at the outcome or study level, or both; state how this information will be used in data synthesis	10
Data synthesis	#15a	Describe criteria under which study data will be quantitatively synthesised	10, 11
Data synthesis	#15b	If data are appropriate for quantitative synthesis, describe planned summary measures, methods of handling data and methods of combining data from studies, including any planned exploration of consistency (such as I2, Kendall's τ)	10, 11

For peer review only

Data synthesis	#15c	Describe any proposed additional analyses (such as sensitivity or subgroup analyses, meta-regression)	10, 11
Data synthesis	#15d	If quantitative synthesis is not appropriate, describe the type of summary planned	n/a
Meta-bias(es)	#16	Specify any planned assessment of meta-bias(es) (such as publication bias across studies, selective reporting within studies)	10, 11
Confidence in cumulative evidence	#17	Describe how the strength of the body of evidence will be assessed (such as GRADE)	11

None The PRISMA-P elaboration and explanation paper is distributed under the terms of the Creative Commons Attribution License CC-BY. This checklist can be completed online using https://www.goodreports.org/, a tool made by the EQUATOR Network in collaboration with Penelope.ai

For peer review only

BMJ Open

Burden of Mental Health Problems Among Pregnant and Post-Partum Women in Sub-Saharan Africa: Systematic Review and Meta-analysis Protocol

Journal:	*BMJ Open*
Manuscript ID	bmjopen-2022-069545.R1
Article Type:	Protocol
Date Submitted by the Author:	21-Apr-2023
Complete List of Authors:	Awini, Elizabeth; Ghana Health Service<br>Agyepong, Irene; Ghana Health Service; Ghana College of Physicians and Surgeons, Faculty of Public Health<br>Owiredu, David; University of Ghana<br>Gyimah, Leveana; Pantang Hospital; Ghana College of Physicians and Surgeons, Faculty of Psychiatry<br>Ashinyo, Mary; Ghana Health Service, Institutional Care Division<br>Yevoo, Lucy; Ghana Health Service<br>Aye, Sorre Grace Emmanuelle Victoire ; Ghana Health Service, Research and Development Division; Ghana College of Physicians and Surgeons, Faculty of Public Health<br>Abbas, Shazra; The University of Melbourne, Nossal Institute for Global Health, Melbourne School of Population and Global Health<br>Cronin de Chavez, Anna; London School of Hygiene and Tropical Medicine Department of Global Health and Development<br>Kane, Sumit; The University of Melbourne, Melbourne School Of Population And Global Health<br>Mirzoev, Tolib; London School of Hygiene & Tropical Medicine, Global Health and Development<br>Danso-Appiah , Anthony ; University of Ghana, Epidemiology and Disease Control; University of Ghana, Centre for Evidence Synthesis and Policy
**Primary Subject Heading**:	Mental health
Secondary Subject Heading:	Epidemiology, Global health, Public health
Keywords:	MENTAL HEALTH, Maternal medicine < OBSTETRICS, PSYCHIATRY

SCHOLARONE™
Manuscripts

Burden of Mental Health Problems Among Pregnant and Post-Partum Women in Sub-Saharan Africa: Systematic Review and Meta-analysis Protocol

Elizabeth Awini[1], Irene Akua Agyepong[1,2], David Owiredu[3], Leveana Gyimah[4,5], Mary Eyram Ashinyo[6], Linda Lucy Yevoo[1], Sorre Grace Emmanuelle Victoire Aye[1,2], Shazra Abbas[7], Anna Cronin de Chavez[8], Sumit Kane[7], Tolib Mirzoev[8], Anthony Danso-Appiah[3,9,*]

Author affiliation

[1]Dodowa Health Research Centre, Research and Development Division, Ghana Health Service, Dodowa, Ghana
[2]Ghana College of Physicians and Surgeons, Faculty of Public Health, Accra, Ghana
[3]Department of Epidemiology and Disease Control, School of Public Health, University of Ghana, Legon, Ghana
[4]Pantang Hospital, Accra, Ghana
[5]Ghana College of Physicians and Surgeons, Faculty of Psychiatry, Accra, Ghana
[6]Institutional Care Division, Ghana Health Service, Accra, Ghana
[7]Nossal Institute for Global Health, Melbourne School of Population and Global Health, The University of Melbourne, Victoria 3010 Australia
[8]Department of Global Health and Development, London School of Hygiene and Tropical Medicine, UK
[9] Centre for Evidence Synthesis and Policy, University of Ghana, Legon, Accra, Ghana

Author email addresses

Elizabeth Awini; awini.elizabeth@gmail.com
Irene Akua Agyepong; irene.agyepong@gmail.com
David Owiredu; evasupreme@gmail.com
Leveana Gyimah; leveana12@gmail.com
Mary Eyram Ashinyo; mary.ashinyo@ghsmail.org
Linda Lucy Yevoo; lyevoo@gmail.com
Sorre Grace Emmanuelle Victoire Aye; graceaye7@gmail.com
Shazra Abbas; shazra.abbas1@unimelb.edu.au
Anna Cronin de Chavez; Anna.Chavez@lshtm.ac.uk
Sumit Kane; sumit.kane@unimelb.edu.au
Tolib Mirzoev; Tolib.Mirzoev@lshtm.ac.uk

***Corresponding author**

Dr Antony Danso-Appiah, DSc, PhD

1. Department of Epidemiology and Disease Control, School of Public Health
   University of Ghana, P. O. Box LG13, Legon, Accra, Ghana
2. UG Centre for Evidence Synthesis and Policy, School of Public Health
   University of Ghana, P. O. Box LG13, Legon, Accra, Ghana

Email: adanso-appiah@ug.edu.gh
tdappiah@yahoo.co.uk

For peer review only - http://bmjopen.bmj.com/site/about/guidelines.xhtml

Tel: +233 269083789
+44 7958721789

Keywords:
Maternal, mental health, mental disorders, mental conditions, perinatal, pregnant women, postpartum women, burden of disease, Sub-Saharan Africa

ORCID IDs
Elizabeth Awini: **0000-0002-1147-985X**

Irene Akua Agyepong: **0000-0002.0193.5882**

David Owiredu: **0000-0002-4503-1850**

Leveana Gyimah: **0000-0003-2323-4718**

Mary Eyram Ashinyo: **0000-0002-8493-9378**

Linda Lucy Yevoo: **0000-0003-2309-2158**

Aye Sorre Grace Emmanuelle Victoire **0000-0001-7435-677X**

Shazra Abbas **0000-0002-5402-0766**

Anna Cronin de Chavez: **0000-0002-4050-4276**

Sumit Kane: **0000-0002-4858-7344**

Tolib Mirzoev: **0000-0003-2959-9187**

Anthony Danso-Appiah: **0000-0003-1747-0060**

Word count

Main Text Word Count: 2689

Tables: 1

For peer review only

For peer review only - http://bmjopen.bmj.com/site/about/guidelines.xhtml

Abstract

Introduction

Pregnancy and postpartum- related mental health problems pose serious public health threat to the society, but worryingly, neglected in Sub-Saharan Africa (SSA). This review will assess the burden and distribution of maternal mental health (MMH) problems in SSA, with the aim to inform the implementation of context sensitive interventions and policies.

Methods and analysis

All relevant databases, grey literature and non-database sources will be searched. PubMed, LILAC, CINAHL, SCOPUS and PsycINFO, Google Scholar, African Index Medicus, HINARI, African Journals Online and IMSEAR will be searched from inception to 31st May 2023, without language restriction. The reference lists of articles will be reviewed, and experts contacted for additional studies missed by our searches. Study selection, data extraction and risk of bias assessment will be done independently by at least two reviewers and any discrepancies will be resolved through discussion between the reviewers. Binary outcomes (prevalence and incidence) of MMH problems will be assessed using pooled proportions, Odds Ratio (OR) or Risk Ratio (RR), and Mean Difference (MD) for continuous outcomes; all will be presented with their 95% confidence intervals (CIs). Heterogeneity will be investigated graphically for overlapping CIs and statistically using the I^2 statistic and where necessary subgroup analyses will be performed. Random-effects model meta-analysis will be conducted when heterogeneity is appreciable, otherwise fixed-effect model will be used. The overall level of evidence will be assessed using GRADE.

Ethics, dissemination and protocol registration

Although no ethical clearance or exemption is needed for a systematic review, this review is part of a larger study on maternal mental health which has received ethical clearance from the Ethics Review Committee of the Ghana Health Service (GHS-ERC 012/03/20). Findings of this study will be disseminated through stakeholder forums, conferences and peer review publications. This systematic review has been registered in PROSPERO- CRD42021269528.

For peer review only - http://bmjopen.bmj.com/site/about/guidelines.xhtml

Strengths and limitations of this study

- This study uses robust methods, best practices and reporting guidelines to attempt to synthesize evidence on the burden of MMH problems in SSA to provide country and regional- specific estimates.
- Screening of articles, data extraction and quality assessment will be done using validated tools by at least two independent reviewers to minimize bias.
- The study uses comprehensive search terms and strategy, and involves relevant electronic databases and non-database sources to attempt to retrieve all potentially relevant studies.
- A possible limitation is, there were no previous data to compare our systematic review to.

Introduction

Maternal mental health (MMH) problems, occurring during pregnancy and postpartum constitute a serious public health problem globally, affecting about 10% of pregnant women and 13% of postpartum mothers[1-3]. A systematic review on global depression among postpartum women estimated prevalence of 17.2% worldwide[4]. In high income countries, prevalence of postpartum depression ranged from 5% to 20%[5]. Data from 2010-2015 Northern Ireland Maternity System indicated that 18.9% of pregnant women reported a history of at least one mental disorder[6]. In the United States, 10% depression in mothers was reported over 12 months[7]. Analysis of Claims data from January to December 2008 involving 38,174 pregnant women in Germany identified at least one mental health problem from four main mental health disorders in 16,639 of the women[8] with somatoform/dissociative disorder being 24.2%, anxiety 16.9%, stress reactions 11.7%, and depression 9.3%. While reliable data are not readily available, existing estimates suggest the burden of MMH problems is relatively higher in low- and middle-income countries (LMICs) with 1 in 4 women reporting depression during pregnancy and 1 in 5 after delivery[9]. A review of evidence from LMICs on pre and postnatal depression reported prevalence of 4.9% to 50%[10] whereas a systematic review involving pre- and postnatal women living in Africa reported prevalence of depression during pregnancy at 11.3% and 18.3 % after delivery[11]. Another review in SSA reported 18.6% post-partum depression[12], with a range from 7% to 50.3%.

The common mental health problems experienced by pregnant and postpartum women are depression and anxiety[13-16]. Depression can be mild, moderate or severe. Mild depression will normally not affect the individual's ability to undertake their day-to-day activities such as self-care and interpersonal relationships whereas moderate to severe episodes can render the mother less capable of undertaking their basic self-care

For peer review only - http://bmjopen.bmj.com/site/about/guidelines.xhtml

and that of their newborn babies which could impact on breastfeeding and bonding[17]. Other maternal mental health disorders are postpartum psychosis, pregnancy and postpartum obsessive-compulsive disorder (OCD), birth-related posttraumatic stress disorder (PTSD), intrusive thoughts and mania, schizophrenia, infanticide, substance use disorder, anorexia nervosa, bulimia, suicidal ideation, bipolar affective disorder, paranoia, psychopathy, neurotic disorders and self-harm[18, 19]. These disorders may develop as a result of experiences associated with childbirth, foetal loss, congenital malformations, intimate partner violence during pregnancy, lack of partner support, history of abuse, unplanned pregnancy, complications in previous pregnancy, higher perceived stress, lower self-esteem[20-24], and many more. Individuals with predisposition to bipolar affective disorders may develop manic episodes from pregnancy-related stress[20]. Severity of maternal mental health disorders can potentially progresses from mild or moderate to severe forms if not identified early and appropriate intervention instituted[25, 26].

Rationale for this systematic review

Mothers with mental health problems may not be able to realize their abilities, work productively or cope with the normal stresses of life[3]. Maternal mental health disorders can have negative effects on both the mother and the child[27]. In severe cases, depressed mothers may have symptoms of a psychosis[4]. Affected mothers often cannot function properly, with some having suicidal tendencies[3]. Evidence shows that maternal mental health problems are associated with negative birth outcomes and may adversely affect cognitive development of the infant[28, 29], nutritional status of their infants[30], and early child wellbeing[31]. There is, however, limited knowledge to inform policy makers on the burden of maternal mental health disorder in SSA to inform on the selection and implementation of locally relevant, feasible, and effective interventions. Maternal mental health is an important health problem which should be given the needed attention but this has often been neglected as a health priority[32-34.] There is much evidence that in LMICs the vast majority of women who experience mental health problems during and after pregnancy do not receive the needed treatment[1, 35]. Although few systematic reviews have investigated mental health problems in SSA, none focused specifically on MMH[36, 37]. The only review that assessed risk factors for antenatal depression, included only studies conducted in Ethiopia[36], and although a systematic review protocol has been registered in PROSPERO[37], it intends to explore the role of MMH disorders and stillbirths.

For peer review only - http://bmjopen.bmj.com/site/about/guidelines.xhtml

This review will assess the burden of MMH problems among pregnant and post-partum women in SSA and attempts to provide robust country and regional estimates of the burden of disease across countries in SSA. Specifically, it will determine the prevalence and incidence and describe the socio-demographic characteristics, general obstetric histories and characteristics of women with MMH problems; assess effects of maternal mental health problems on birth outcomes; and analyze for differences in the burden of mental health problems among pregnant and post-partum women between rural and urban settings. The review will answer the following specific questions: 1) What is the magnitude of MMH problems among pregnant and post-partum women living in SSA? 2) What are the socio-demographic characteristics of pregnant and post-partum women with mental health problems in SSA? 3) What are the general obstetric histories and characteristics of pregnant and post-partum women in SSA with mental health problems? 4) Is there any difference in the burden of pregnant and post-partum women with mental health problems between rural and urban settings? and 6) Are there any documented effects of birth outcomes of pregnant women with mental health problems?

Review methods

This review protocol has been prepared following the Preferred Reporting Items for Systematic Review and Meta-Analysis extension for protocols (PRISMA-P)[38] (Supplemental file 1) and the PRISMA flow diagram (Supplemental file 2). The full review will be prepared in line with the Preferred Reporting Items for Systematic Review and Meta-Analysis (PRISMA). The full review is expected to start on 1st May 2023, analysis 1st September and completed by 30th November 2023.

Patient and public involvement

The review questions and outcome measures have been developed collaboratively with the relevant patient and consumer involvement and informed by their priorities, experience, and preferences in line with GRIPP2 reporting checklists. The review findings will be shared with relevant wider patient communities who will also be involved in the results dissemination.

Criteria for considering studies for this review

Types of studies

Any study conducted in SSA that assessed burden of maternal mental health problems among pregnant and post-partum women will be eligible for inclusion. This review is not an intervention effectiveness review and randomized controlled trials (RCTs) are not the focus but if a RCT reported baseline

For peer review only

For peer review only - http://bmjopen.bmj.com/site/about/guidelines.xhtml

number of cases and used a well-defined sample to serve as the denominator to allow the calculation of proportion/prevalence/incidence in pregnant or postpartum women, such an RCT will be eligible for inclusion. Reviews will not be eligible for inclusion. However, we will go through the reviews to identify potentially eligible studies missed by our searches. If the study is a global review having, for example, SSA or sub-regional sub-set we will extract the studies conducted in SSA for inclusion in this review. If the study reported a country or regional estimate without a well-defined sample (representative sample or sub-sample of the source population), it will not be eligible for inclusion. In cases where the results of a multi-country study have been lumped together and there is no way of disaggregating the data, such studies will not be included. Case studies and case series (these are atypical and not representative of the source population), commentaries or opinions, will not be eligible for inclusion.

Participants

Pregnant and post-partum (postpartum is defined as up to 12 months after delivery) women living in SSA, diagnosed of any mental health disorder (depression, anxiety, postpartum psychosis, bipolar disorders, substance misuse disorders, dysthymia, obsessive-compulsive disorder, posttraumatic stress disorder, schizophrenia, infanticide, suicidal ideation, paranoia, psychopathy, neurosis/neurotic disorders, self-harm, anorexia nervosa, bulimia, etc.) will be eligible for inclusion. The tool or criteria for diagnosis should be stated, for example, standard operational diagnostic criteria such as Research Diagnostic Criteria (RDC)[39], the 10th edition of the International Classification of Diseases (ICD-10)[40] or Diagnostic and Statistical Manual of Mental Disorders (DSM-V)[41]. Pregnant or post-partum women whose diagnosis of mental health disorder could not be confirmed will be excluded.

Interventions

This systematic review is not intervention review.

Comparison

This is non-comparative review but where outcomes or variables permit comparison, we will attempt to compare.

Outcomes

Primary outcomes

- Burden of MMH problems measured as prevalence, incidence etc.
- Birth outcomes (maternal and foetal outcomes) up to 12 months postpartum
- Type of MMH conditions (for diagnosed cases)

For peer review only - http://bmjopen.bmj.com/site/about/guidelines.xhtml

Secondary outcomes

- Proportion of MMH conditions captured in the community
- Predisposition socio-demographic characteristics of pregnant and post-partum women with mental health problems
- Predisposition obstetric histories and characteristics of pregnant and post-partum women in SSA with mental health problems
- Psychiatric predisposition (pre-existing mental health issues triggered during or after pregnancy).
- Burden of mental health problems among pregnant and post-partum women between rural and urban settings

Search strategy

We will search the following electronic databases: PubMed, PsycINFO, LILAC and CINAHL from inception to 31st May 2023 without language restriction and using the search terms in Table 1. We will also search Google Scholar, African Index Medicus, HINARI, African Journals Online, IMSEAR and relevant preprint repositories. Grey literature including dissertations and conference proceedings will be searched. Reference list of retrieved articles will be reviewed, and experts in the field of MMH will be contacted for studies not captured by our searches (see Table 1 for search strategy developed for PubMed).

For peer review only

For peer review only - http://bmjopen.bmj.com/site/about/guidelines.xhtml

Table 1 Search Strategy for PubMed (to be adapted for the other databases)

Search	Query	Results
#1	Search: **((((((((((((((((((((((("mental health"[Title/Abstract]) OR ("mental disorder"[Title/Abstract])) OR ("mental illness"[Title/Abstract])) OR ("mental problem"[Title/Abstract])) OR ("chronic mental illness"[Title/Abstract])) OR ("psychiatric illness"[Title/Abstract])) OR ("chronic psychiatric illness"[Title/Abstract])) OR ("chronic insanity"[Title/Abstract])) OR (insanity[Title/Abstract])) OR ("chronic mental disorder"[Title/Abstract])) OR ("dementia praecox"[Title/Abstract])) OR (schizophrenia[Title/Abstract])) OR (psychoses[Title/Abstract])) OR (psychosis[Title/Abstract])) OR ("schizophrenic psychosis"[Title/Abstract])) OR (depression[Title/Abstract])) OR ("mental sickness"[Title/Abstract])) OR ("mental disease"[Title/Abstract])) OR (maladjustment[Title/Abstract])) OR ("emotional disorder"[Title/Abstract])) OR ("nervous disorder"[Title/Abstract])) OR ("nervous breakdown"[Title/Abstract])) OR (neurosis[Title/Abstract])) OR ("neurotic disorder"[Title/Abstract])) OR (psychopathy[Title/Abstract])) OR (paranoia[Title/Abstract])**	
#2	Search: **(((((((((((("pregnant women"[Title/Abstract]) OR (prepartum[Title/Abstract])) OR (peripartum[Title/Abstract])) OR (perinatal[Title/Abstract])) OR (prenatal[Title/Abstract])) OR (antenatal[Title/Abstract])) OR ("during pregnancy"[Title/Abstract])) OR (postpartum[Title/Abstract])) OR (maternal[Title/Abstract])) OR (maternity[Title/Abstract])) OR (parturient[Title/Abstract])) OR (antepartum[Title/Abstract])) OR ("post-delivery"[Title/Abstract])) OR (puerperium[Title/Abstract])**	
#3	Search: **(#1) AND (#2)**	
#4	Search: **((("sub-Saharan Africa") OR (SSA)) OR (Angola)) OR (Benin)) OR (Botswana)) OR ("Burkina Faso")) OR (Burundi)) OR (Cameroon)) OR ("Cape Verde")) OR ("Central African Republic")) OR (Chad)) OR (Comoros)) OR (Congo)) OR ("Cote d'Ivoire")) OR (Djibouti)) OR ("Equatorial Guinea")) OR (Ethiopia)) OR (Gabon)) OR ("The Gambia")) OR (Ghana)) OR (Guinea)) OR ("Guinea-Bissau")) OR (Kenya)) OR (Lesotho)) OR (Liberia)) OR (Madagascar)) OR (Malawi)) OR (Mali)) OR (Mauritania)) OR (Mauritius)) OR (Mozambique)) OR (Namibia)) OR (Niger)) OR (Nigeria)) OR (Rwanda)) OR ("Sao Tome and Principe")) OR (Senegal)) OR (Seychelles)) OR ("Sierra Leone")) OR (Somalia)) OR ("South Africa")) OR (Sudan)) OR (Swaziland)) OR (Tanzania)) OR (Togo)) OR (Uganda)) OR (Zaire)) OR (Zambia)) OR (Zimbabwe)**	
#5	Search: **(#3) AND (#4)**	

The search strategy will be adapted as appropriate for other databases. All searches will be re-run just before the final analyses and any further eligible studies identified will be included.

Study selection

The search output will be managed, collated and de-duplicated using EndNote. The deduplicated articles will be exported to Rayyan[42] for screening and selection. At least two reviewers will screen

For peer review only - http://bmjopen.bmj.com/site/about/guidelines.xhtml

titles and abstracts of identified studies independently using pre-specified and piloted eligibility flowchart (Figure 1). The full text of potentially relevant studies will also be reviewed independently for inclusion. The PRISMA flow diagram will be used to document the flow of studies and reasons for exclusion. Discrepancies will be resolved through discussion between the reviewers.

Data extraction and management

A validated data extraction sheet adapted from the Cochrane Collaboration (Supplemental file 3) will be used by the reviewers to independently extract relevant data. Data to be extracted include characteristics of the studies such as year study was conducted, year study was published (for published studies), country where study was conducted, study design and sample size; socio-demographic characteristics of the participants (age, setting, socio-economic status, level of education and occupation); obstetric factors (parity, maternal age, age at first delivery, history of miscarriage, history of stillbirth), mental health conditions (depression, anxiety, postpartum psychosis, dysthymia/persistent depressive disorder, pregnancy and postpartum obsessive-compulsive disorder (OCD), birth- related posttraumatic stress disorder (PTSD), intrusive thoughts and mania, schizophrenia, infanticide, substance use disorder, anorexia nervosa, bulimia, suicidal ideation, bipolar affective disorder, paranoia, psychopathy, neurotic disorders and self-harm); and quality domains for the assessment of quality of the included studies for risk of bias. Data on burden of the disease (such as prevalence, incidence and duration of the MMH problem) will be extracted. The corresponding authors of the primary studies will be contacted for missing data or unclear information. Where it is not possible to obtain the missing information, data will be analyzed based on those with complete outcome data and the amount of data missing with reasons will be provided. If necessary, data will be coded and recoded before use in the analysis. The extracted data will be verified independently and any disagreement will be resolved through discussion.

Assessment of quality of the included studies

At least two reviewers will assess quality of the included studies for risk of bias (methodology and reporting) independently using the appropriate risk of bias assessment tools. Since this review is not focusing on randomized controlled trials (RCTs), the domains on the Cochrane Risk of Bias (ROB) tool[43] will not be covered fully. Selective outcome and analysis reporting bias (which occurs when studies with positive and significant results are likely to be reported or published) are the domains to be considered on the Cochrane ROB tool. The Cochrane criteria for reporting risk of bias in the included studies will be used to assess pre-specified outcomes of interest. The risk

For peer review only - http://bmjopen.bmj.com/site/about/guidelines.xhtml

of bias will be rated as "low" if protocol of the study is available and all pre-specified outcomes of interest as specified in the protocol, or the protocol is not available but it is clear that all pre-specified and expected outcomes of interest have been reported. The risk of bias will be rated as "high" for a study if outcomes are not reported as pre-specified or expected and "unclear" when there is not enough information to make clear judgement. Other pre-specified biases pertaining to the methods, source of funding etc will be assessed and rated. The Risk of Bias Tool for Prevalence studies will be assessed using the tool by Hoy et al[44] (Supplemental file 4) on four domains: selection bias, non-response bias, measurement bias and bias related to data analysis for prevalence studies. Each risk of bias domain will be graded as 'low risk', 'high risk', and 'unclear' risk of bias. Any disagreements will be resolved through discussion between the reviewers, and if necessary, a third reviewer will be consulted.

Data analysis

Binary outcomes will be assessed using Odds Ratio (OR) or Risk Ratio (RR), and for continuous data, we will use Mean difference (MD) or Standardized Mean Difference (SMD) for means that used different scales. Meta-Analysis, the statistical component of systematic review, will be used to combine study outcomes. Odds Ratio, RR and MD of the individual studies will be pooled and presented with their 95% confidence interval (CI). Random-effects model will be used in the meta-analysis when heterogeneity is high, otherwise fixed-effect model will be used. Descriptive statistics will be used to describe proportions (prevalence and incidence).

Heterogeneity and subgroup analysis

Heterogeneity arises because of variation in the study design, characteristics of participants or outcomes between or within studies[45]. Heterogeneity will be investigated both graphically and statistically. The I^2 statistic which describes the percentage of variability that is due to heterogeneity rather than chance will be estimated. The I^2 is classified into four levels: 0%, 1-29%, 30-59% and 60-100%[46] and I^2>50% indicates significant heterogeneity[47]. Subgroup analysis which is used to estimate an effect of indicator within each subgroup or subset will be used to address heterogeneity due to variation in effects in each subgroup mixed effect model. Thus, the burden of MMH problems will be estimated separated for urban and rural and for each age group. Subgroup analysis will also be done for gestational age and postpartum period. If we find sufficient number of studies, subgroup analysis will also be based on perinatal mental disorder type, study design, parity and location.

For peer review only - http://bmjopen.bmj.com/site/about/guidelines.xhtml

Grading the evidence

The overall evidence of the systematic review will be graded using Grading of Recommendations Assessment, Development and Evaluation (GRADE)[48] (Available from guidelinedevelopment.org/handbook). The GRADE system assesses the following domains: risk of bias, imprecision, inconsistency, indirectness and publication bias, and classifies the quality of evidence as high, moderate, low, and very low. If evidence from a study is graded high quality, it implies further research is very unlikely to change the confidence in the estimate of effect while a grading of very low quality implies an estimate of effect is very doubtful. This protocol has been registered on the International Prospective Register of Systematic Reviews (PROSPERO) with registration **ID CRD42021269528.**

Ethical approval and dissemination

Although no ethical clearance or exemption is needed for a systematic review, this review is part of a larger study on maternal mental health that involves primary data collection and which has received ethical clearance from the ethics review committee of the Ghana Health Service (GHS-ERC 012/03/20). The results of the review will be presented to stakeholders (policymakers and practitioners), It will also be disseminated through conferences and peer review publications.

Author contributions

Protocol conceptualization and design: EA, IAA, SK, TM and ADA

Drafting the work or revising it critically for important intellectual content: EA, IAA, DO, LG, MEA, LLY, SGEVA, SA, ACC, SK, TM and ADA

Acquisition, analysis, or interpretation of data: EA, IAA, DO, LG, MEA, LLY, SGEVA, SA, ACC, SK, TM and ADA

Agreement to be accountable for accuracy or integrity of all aspects of the work: EA, IAA, DO, LG, MEA, LLY, SGEVA, SA, ACC, SK, TM and ADA

Final approval of the version to be published: EA, IAA, DO, LG, MEA, LLY, SGEVA, SA, ACC, SK, TM and ADA

Supervised this work: ADA

Acknowledgments

We thank Dr. Caleb Othieno, University of Nairobi, Kenya, Mrs. Ruth Owusu-Antwi, Komfo Anokye Teaching Hospital, Kwame Nkrumah University of Science and Technology, Kumasi, Ghana and Stephanie Catsaros, Université Paris Cité, France for peer-reviewing this manuscript

For peer review only - http://bmjopen.bmj.com/site/about/guidelines.xhtml

and providing very useful comments. This systematic review was part of capacity building initiative by the UG Centre for Evidence Synthesis and Policy (UGCESP), Africa Communities of Evidence Synthesis and Translation (ACEST) and the RESPONSE Project that are jointly training experts in evidence synthesis and translation across Low and Middle-Income Countries (LMICs).

Funding Statement

This review is part of a wider RESPONSE study, which received funding from the Joint MRC/ESRC/DFID/Wellcome Health Systems Research Initiative (grant ref: MR/T023481/1). The views expressed in this publication are those of the author(s) and not necessarily those of the funders.

Competing of interest: None declared.

For peer review only

For peer review only - http://bmjopen.bmj.com/site/about/guidelines.xhtml

References

1. Kathree T, Selohilwe OM, Bhana A, Petersen I. Perceptions of postnatal depression and health care needs in a South African sample: the "mental" in maternal health care. *BMC Women's Health.* 2014;14(1):1-11.
2. McCauley M, Brown A, Ofosu B, Van Den Broek N. "I just wish it becomes part of routine care": healthcare providers' knowledge, attitudes and perceptions of screening for maternal mental health during and after pregnancy: a qualitative study. *BMC psychiatry.* 2019;19(1):1-8.
3. WHO. WHO | Maternal mental health. Available at: https://www.who.int/mental_health/maternal-child/maternal_mental_health/en/. Accessed 20 December 2020, 2020.
4. Wang Z, Liu J, Shuai H, et al. Mapping global prevalence of depression among postpartum women. *Translational psychiatry.* 2021;11(1):1-13.
5. Nilaweera I, Doran F, Fisher J. Prevalence, nature and determinants of postpartum mental health problems among women who have migrated from South Asian to high-income countries: a systematic review of the evidence. *Journal of Affective Disorders.* 2014;166:213-226.
6. Mongan D, Lynch J, Hanna D, et al. Prevalence of self-reported mental disorders in pregnancy and associations with adverse neonatal outcomes: a population-based cross-sectional study. *BMC pregnancy and childbirth.* 2019;19(1):1-10.
7. Ertel KA, Rich-Edwards JW, Koenen KC. Maternal depression in the United States: Nationally representative rates and risks. *Journal of Women's Health.* 2011;20(11):1609-1617.
8. Wallwiener S, Goetz M, Lanfer A, et al. Epidemiology of mental disorders during pregnancy and link to birth outcome: a large-scale retrospective observational database study including 38,000 pregnancies. *Archives of gynecology and obstetrics.* 2019;299(3):755-763.
9. Gelaye B, Rondon MB, Araya R, Williams MA. Epidemiology of maternal depression, risk factors, and child outcomes in low-income and middle-income countries. *The Lancet Psychiatry.* 2016;3(10):973-982.
10. Parsons CE, Young KS, Rochat TJ, Kringelbach ML, Stein A. Postnatal depression and its effects on child development: a review of evidence from low-and middle-income countries. *British medical bulletin.* 2012;101(1):57-79.
11. Sawyer A, Ayers S, Smith H. Pre-and postnatal psychological wellbeing in Africa: a systematic review. *Journal of affective disorders.* 2010;123(1-3):17-29.
12. Woldeyohannes D, Tekalegn Y, Sahiledengle B, Ermias D, Ejajo T, Mwanri L. Effect of postpartum depression on exclusive breast-feeding practices in sub-Saharan Africa countries: a systematic review and meta-analysis. *BMC Pregnancy and Childbirth.* 2021;21(1):1-10.
13. Atif N, Lovell K, Rahman A. Maternal mental health: The missing "m" in the global maternal and child health agenda. Paper presented at: Seminars in perinatology, 2015.
14. Banti S, Mauri M, Oppo A, et al. From the third month of pregnancy to 1 year postpartum. Prevalence, incidence, recurrence, and new onset of depression. Results from the Perinatal Depression–Research & Screening Unit study. *Comprehensive psychiatry.* 2011;52(4):343-351.
15. Garthus-Niegel S, von Soest T, Knoph C, Simonsen TB, Torgersen L, Eberhard-Gran M. The influence of women's preferences and actual mode of delivery on post-traumatic stress symptoms following childbirth: a population-based, longitudinal study. *BMC pregnancy and childbirth.* 2014;14(1):1-10.
16. Falah-Hassani K, Shiri R, Dennis C-L. Prevalence and risk factors for comorbid postpartum depressive symptomatology and anxiety. *Journal of affective disorders.* 2016;198:142-147.

For peer review only - http://bmjopen.bmj.com/site/about/guidelines.xhtml

17. Park LT, Zarate Jr CA. Depression in the primary care setting. *New England Journal of Medicine.* 2019;380(6):559-568.

18. Bauer A, Parsonage M, Knapp M, Iemmi V, Adelaja B, Hogg S. The costs of perinatal mental health problems. *London: Centre for Mental Health and London School of Economics.* 2014.

19. Collardeau F, Corbyn B, Abramowitz J, Janssen PA, Woody S, Fairbrother N. Maternal unwanted and intrusive thoughts of infant-related harm, obsessive-compulsive disorder and depression in the perinatal period: study protocol. *BMC psychiatry.* 2019;19(1):1-15.

20. Biaggi A, Conroy S, Pawlby S, Pariante CM. Identifying the women at risk of antenatal anxiety and depression: a systematic review. *Journal of affective disorders.* 2016;191:62-77.

21. Silva MMdJ, Nogueira DA, Clapis MJ, Leite EPRC. Anxiety in pregnancy: prevalence and associated factors. *Revista da Escola de Enfermagem da USP.* 2017;51.

22. Ogbo FA, Eastwood J, Hendry A, et al. Determinants of antenatal depression and postnatal depression in Australia. *BMC psychiatry.* 2018;18(1):1-11.

23. Milgrom J, Hirshler Y, Reece J, Holt C, Gemmill AW. Social support—a protective factor for depressed perinatal women? *International journal of environmental research and public health.* 2019;16(8):1426.

24. Koh M, Ahn S, Kim J, Park S, Oh J. Pregnant women's antenatal depression and influencing factors. *Korean Journal of Women Health Nursing.* 2019;25(1):112-123.

25. Sadock BSV. Postpartum Psychiatric Syndromes. *Comprehensive Textbook of Psychiatry.* Vol 1. 7 ed: Lippincott & Wilkins Publishers.; 2000:992–1053.

26. Semple D, & Smyth, R. Disorders related to childbirth. *Oxford Handbook of Psychiatry*. 3rd ed. Oxford: Oxford University Press; 2013:470–472.

27. O'hara MW, McCabe JE. Postpartum depression: current status and future directions. *Annu Rev Clin Psychol.* 2013;9(1):379-407.

28. Tran TD, Biggs B-A, Tran T, et al. Impact on infants' cognitive development of antenatal exposure to iron deficiency disorder and common mental disorders. *Plos one.* 2013;8(9):e74876.

29. Paschetta E, Berrisford G, Coccia F, et al. Perinatal psychiatric disorders: an overview. *American Journal of Obstetrics and Gynecology.* 2014;210(6):501-509. e506.

30. Hassan BK, Werneck GL, Hasselmann MH. Maternal mental health and nutritional status of six-month-old infants. *Revista de saude publica.* 2016;50:7.

31. MacGinty RP, Lesosky M, Barnett W, Stein DJ, Zar HJ. Associations between maternal mental health and early child wheezing in a South African birth cohort. *Pediatric pulmonology.* 2018;53(6):741-754.

32. O'Mahony JM, Donnelly TT. How does gender influence immigrant and refugee women's postpartum depression help-seeking experiences? *Journal of psychiatric and mental health nursing.* 2013;20(8):714-725.

33. Bagadia A, Chandra PS. Starting the conversation-Integrating mental health into maternal health care in India. *The Indian journal of medical research.* 2017;145(3):267.

34. Cheung FK, Snowden LR. Community mental health and ethnic minority populations. *Community Mental Health Journal.* 1990;26(3):277-291.

35. Fonseca A, Canavarro MC. Women's intentions of informal and formal help-seeking for mental health problems during the perinatal period: The role of perceived encouragement from the partner. *Midwifery.* 2017;50:78-85.

36. Getinet W, Amare T, Boru B, Shumet S, Worku W, Azale T. Prevalence and risk factors for antenatal depression in Ethiopia: systematic review. *Depression research and treatment.* 2018;2018.

For peer review only - http://bmjopen.bmj.com/site/about/guidelines.xhtml

37. Adane AA, Bailey HD, Marriott R, et al. Role of maternal mental health disorders on stillbirth and infant mortality risk: a protocol for a systematic review and meta-analysis. *BMJ open.* 2020;10(5):e036280.

38. Moher D, Shamseer L, Clarke M, et al. Preferred reporting items for systematic review and meta-analysis protocols (PRISMA-P) 2015 statement. *Systematic reviews.* 2015;4(1):1-9.

39. Spitzer RL EJ, Robins E. Research Diagnostic Criteria: Rationale and Reliability. *Arch Gen Psychiatry.* 1978;35(6):773–782.

40. WHO. The ICD-10 Classification of Mental and Behavioural Disorders. *Clinical description and diagnostic guidelines*; 1993.

41. Nuckols CC, Nuckols CC. The diagnostic and statistical manual of mental disorders, (DSM-5). *Philadelphia: American Psychiatric Association.* 2013.

42. Harrison H, Griffin SJ, Kuhn I, Usher-Smith JA. Software tools to support title and abstract screening for systematic reviews in healthcare: an evaluation. *BMC medical research methodology.* 2020;20(1):1-12.

43. Higgins JP, Altman DG, Gøtzsche PC, et al. The Cochrane Collaboration's tool for assessing risk of bias in randomised trials. *Bmj.* 2011;343.

44. Hoy D, Brooks P, Woolf A, et al. Assessing risk of bias in prevalence studies: modification of an existing tool and evidence of interrater agreement. *Journal of clinical epidemiology.* 2012;65(9):934-939.

45. Spineli LM, Pandis N. Exploring heterogeneity in meta-analysis: Subgroup analysis. Part 1. *American journal of orthodontics and dentofacial orthopedics.* 2020;158(2):302-304. e301.

46. Koletsi D, Fleming PS, Michelaki I, Pandis N. Heterogeneity in Cochrane and non-Cochrane meta-analyses in orthodontics. *Journal of dentistry.* 2018;74:90-94.

47. Chang TH, Chou CC, Chang LY. Effect of obesity and body mass index on coronavirus disease 2019 severity: a systematic review and meta-analysis. *Obesity Reviews.* 2020;21(11):e13089.

48. Schünemann H. *The GRADE handbook*: Cochrane Collaboration; 2013.

For peer review only - http://bmjopen.bmj.com/site/about/guidelines.xhtml

Figure Study selection flowchart

For peer review only

For peer review only - http://bmjopen.bmj.com/site/about/guidelines.xhtml

Figure 1 Study selection flowchart

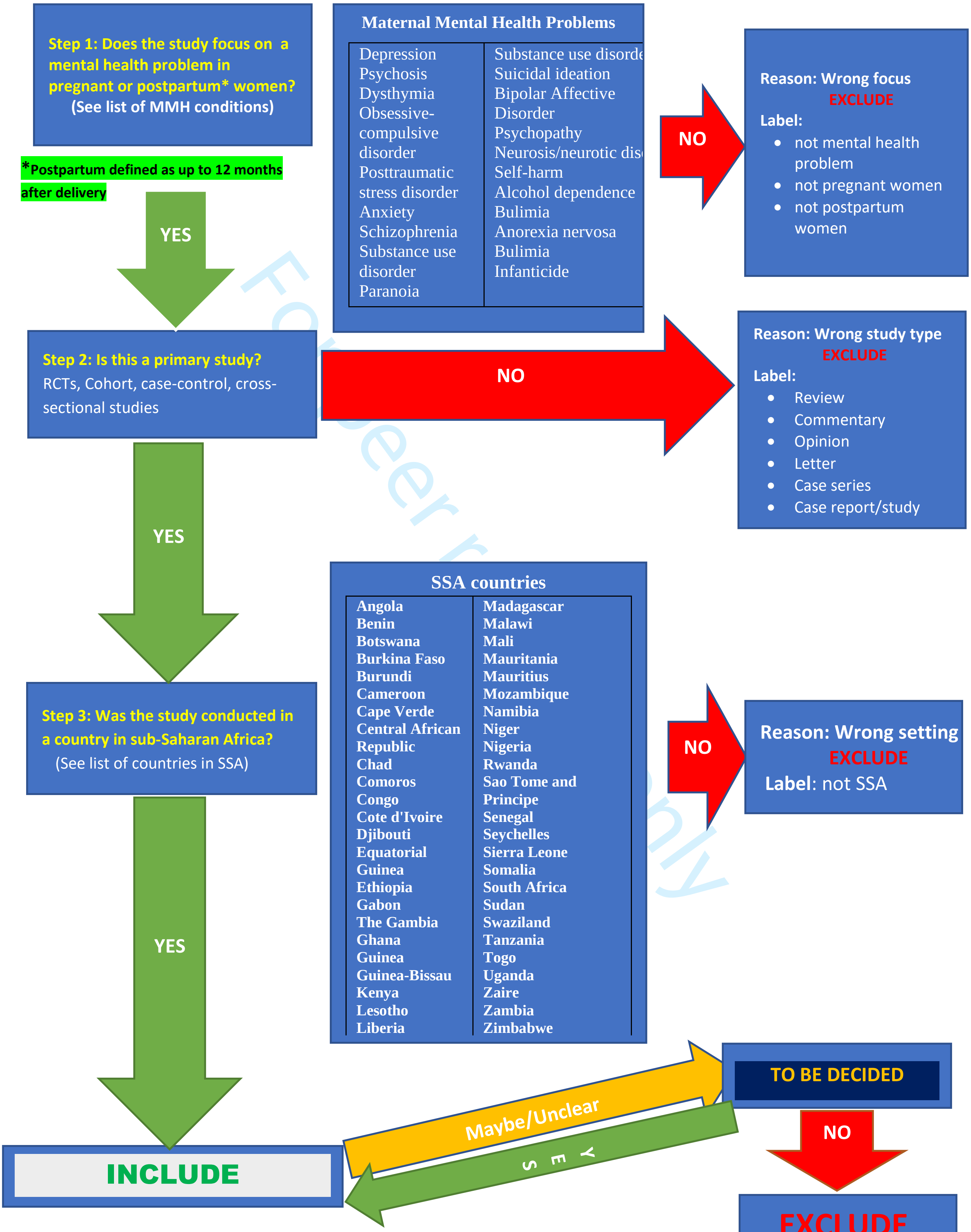

For peer review only

Supplemental file 1 Preferred Reporting Items for Systematic review and Meta-Analysis Protocols (PRISMA-P) checklist*

Section and topic	Item No	Checklist item
**ADMINISTRATIVE INFORMATION**		
Title:		
Identification	1a	Identify the report as a protocol of a systematic review
Update	1b	If the protocol is for an update of a previous systematic review, identify as such
Registration	2	If registered, provide the name of the registry (such as PROSPERO) and registration number
Authors:		
Contact	3a	Provide name, institutional affiliation, e-mail address of all protocol authors; provide physical mailing address of corresponding author
Contributions	3b	Describe contributions of protocol authors and identify the guarantor of the review
Amendments	4	If the protocol represents an amendment of a previously completed or published protocol, identify as such and list changes; otherwise, state plan for documenting important protocol amendments
Support:		
Sources	5a	Indicate sources of financial or other support for the review
Sponsor	5b	Provide name for the review funder and/or sponsor
Role of sponsor or funder	5c	Describe roles of funder(s), sponsor(s), and/or institution(s), if any, in developing the protocol
**INTRODUCTION**		
Rationale	6	Describe the rationale for the review in the context of what is already known
Objectives	7	Provide an explicit statement of the question(s) the review will address with reference to participants, interventions, comparators, and outcomes (PICO)
**METHODS**		
Eligibility criteria	8	Specify the study characteristics (such as PICO, study design, setting, time frame) and report characteristics (such as years considered, language, publication status) to be used as criteria for eligibility for the review
Information sources	9	Describe all intended information sources (such as electronic databases, contact with study authors, trial registers or other grey literature sources) with planned dates of coverage
Search strategy	10	Present draft of search strategy to be used for at least one electronic database, including planned limits, such that it could be repeated
Study records:		
Data management	11a	Describe the mechanism(s) that will be used to manage records and data throughout the review
Selection process	11b	State the process that will be used for selecting studies (such as two independent reviewers) through each phase of the review (that is, screening, eligibility and inclusion in meta-analysis)
Data collection process	11c	Describe planned method of extracting data from reports (such as piloting forms, done independently, in duplicate), any processes for obtaining and confirming data from investigators
Data items	12	List and define all variables for which data will be sought (such as PICO items, funding sources), any pre-planned data assumptions and simplifications
Outcomes and prioritization	13	List and define all outcomes for which data will be sought, including prioritization of main and additional outcomes, with rationale

For peer review only

Risk of bias in individual studies	14	Describe anticipated methods for assessing risk of bias of individual studies, including whether this will be done at the outcome or study level, or both; state how this information will be used in data synthesis
Data synthesis	15a	Describe criteria under which study data will be quantitatively synthesized
	15b	If data are appropriate for quantitative synthesis, describe planned summary measures, methods of handling data and methods of combining data from studies, including any planned exploration of consistency (such as I^2, Kendall's τ)
	15c	Describe any proposed additional analyses (such as sensitivity or subgroup analyses, meta-regression)
	15d	If quantitative synthesis is not appropriate, describe the type of summary planned
Meta-bias(es)	16	Specify any planned assessment of meta-bias(es) (such as publication bias across studies, selective reporting within studies)
Confidence in cumulative evidence	17	Describe how the strength of the body of evidence will be assessed (such as GRADE)

* It is strongly recommended that this checklist be read in conjunction with the PRISMA-P Explanation and Elaboration (cite when available) for important clarification on the items. Amendments to a review protocol should be tracked and dated. The copyright for PRISMA-P (including checklist) is held by the PRISMA-P Group and is distributed under a Creative Commons Attribution Licence 4.0.

For peer review only

Supplemental file 2 PRISMA flow diagram

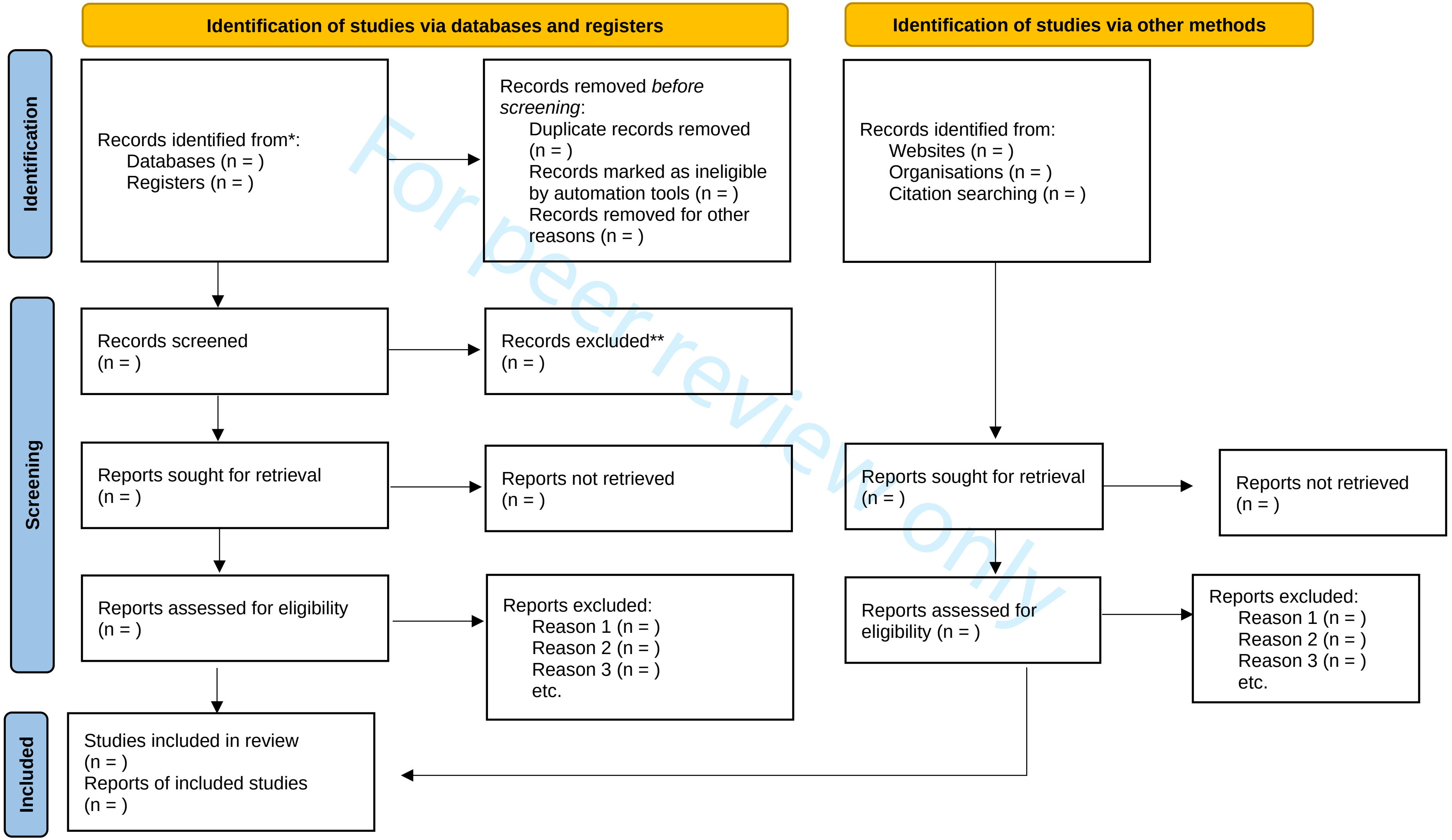

For peer review only

Supplemental file 3 Data extraction sheet (adapted from Cochrane)

**1. General Information**		
1. **Date form completed** *(dd/mm/yyyy)*		
2. **Name/ID of person extracting data**		
3. **Report title** *(title of paper/ abstract/ report that data are extracted from)*		
4. **Publication type** *(e.g. full report, abstract, letter)*		
5. **Study ID** *(e.g. 01 plus surname of first author and year first full report of study was published e.g. Adjuik 2018)*		
6. **Country in which the study conducted**		
7. **Study funding source** *(including role of funders)*		
8. **Possible conflicts of interest** *(for study authors e.g. not reported)*		
**9. Study Characteristics**	**Review Inclusion Criteria** *(as defined in the Protocol)*	**Location in Text** *(page#)*
10. **Type of study**		
11. **Population description**		
12. **Focused diseases / conditions**	*Maternal mental disorders*	
13. **Types of outcome measures**	*Prevalence/incidence/risk ratios, odds ratios/mean difference/proportions*	
14. **Population description** *(from which study participants are drawn)*		
15.. **Source/setting of the population** *(e.g. urban, rural)*		
16. **Method/s of recruitment of participants**		
17. **Aim of study**		
18. **Design** *(e.g. cross-sectional study, cohort study, case-control study etc)*		
19. **Sampling technique** *(e.g. random or convenience)*		
20. **Study start date**		

For peer review only

21. **Study End date/duration** *(if any cohort)*		
22. **Notes:**		
23. **Total number of participants/Sample size**		
24. **Age group**		
25. **Parity**		
26. **Status of mother**	Pregnant, Post-partum	
27. **Outcomes** *(physical/observation examination: who examined?)*		
28. **Self-reported reported outcomes** *(detected by questionnaire/Tools: validated or non-validated?)*		
29. **Outcome names** *(depression, anxiety, psychosis, Birth Related Posttraumatic Stress Disorder, etc)*		
30. **Time points measured** *(report the start month/year/specify whether from start and end of intervention)*		
31. **Time points reported**		
37. **Outcome definition** *(e.g. whether standard case definition used)*		
32. **Type of measurement (***Percentage/Odds ratio/Risk ratio)*		
33. **Is outcome/tool validated?** *(Yes/No/Unclear/Not mentioned)*		
34. **Subgroup** *(if any, e.g. age-specific prevalence reporting)*		
35. **Results**		
36. **Response/non-response rate**		
37. **Any other results reported**		
38. **Unit of analysis** *(e.g. by individuals)*		
39. **Statistical methods used and appropriateness of these methods** *(e.g. proportion/%s, RR/OR)*		

For peer review only

40. **Whether results weighted?** *(e.g. Yes/No)*		
41. **Any other results reported**		
42. **Unit of analysis** *(e.g. by individuals)*		
43. **Statistical methods used and appropriateness of these methods** *(e.g. proportion/%s, RR/OR)*		
44. **All systematic and random error adjusted?** *(e.g. confounding, effect medication etc.)*		
45. **Strength of study**		
46. **Limitation**		
47. **Strategies to overcome the limitation**		
48. **Key conclusions of study authors**		
49. **Notes:**		

For peer review only

Supplemental file 4 Quality assessment checklist for prevalence studies (adapted from Hoy et al., 2012)

Name of author(s):

Year of publication:

Study title:

Risk of bias items	Risk of bias levels	Points scored
1.Was the study's target population a close representation of the national population in relation to relevant variables, e.g. age, sex, occupation?	**Yes** (**LOW RISK**): The study's target population was a close representation of the national population.	0
	**No** (**HIGH RISK**): The study's target population was clearly NOT representative of the national population.	1
2. Was the sampling frame a true or close representation of the target population?	**Yes** (**LOW RISK**): The sampling frame was a true or close representation of the target population.	0
	**No** (**HIGH RISK**): The sampling frame was NOT a true or close representation of the target population.	1
3.Was some form of random selection used to select the sample, OR, was a census undertaken?	**Yes** (**LOW RISK**): A census was undertaken, OR, some form of random selection was used to select the sample (e.g. simple random sampling, stratified random sampling, cluster sampling, systematic sampling).	0
	**No** (**HIGH RISK**): A census was NOT undertaken, AND some form of random selection was NOT used to select the sample.	1
4. Was the likelihood of non-response bias minimal?	**Yes** (**LOW RISK**): The response rate for the study was ≥75%, OR, an analysis was performed that showed no significant difference in relevant demographic characteristics between responders and non- responders	0
	**No** (**HIGH RISK**): The response rate was <75%, and if any analysis comparing responders and non-responders was done, it showed a significant difference in relevant demographic characteristics between responders and non-responders	1
5. Were data collected directly from the subjects (as opposed to a proxy)?	**Yes** (**LOW RISK**): All data were collected directly from the subjects.	0
	**No** (**HIGH RISK**): In some instances, data were collected from a proxy.	1

For peer review only

6.Was an acceptable case definition used in the study?	**Yes (LOW RISK)**: An acceptable case definition was used.	0
	**No (HIGH RISK)**: An acceptable case definition was NOT used	1
7. Was the study instrument that measured the parameter of interest (e.g. prevalence of low back pain) shown to have reliability and validity (if necessary)?	**Yes (LOW RISK)**: The study instrument had been shown to have reliability and validity (if this was necessary), e.g. test-re- test, piloting, validation in a previous study, etc.	0
	**No (HIGH RISK)**: The study instrument had NOT been shown to have reliability or validity (if this was necessary).	1
8. Was the same mode of data collection used for all subjects?	**Yes (LOW RISK)**: The same mode of data collection was used for all subjects.	0
	**No (HIGH RISK)**: The same mode of data collection was NOT used for all subjects.	1
9.Were the numerator(s) and denominator(s) for the parameter of interest appropriate	**Yes (LOW RISK)**: The paper presented appropriate numerator(s) AND denominator(s) for the parameter of interest (e.g. the prevalence of low back pain).	0
	**No (HIGH RISK)**: The paper did present numerator(s) AND denominator(s) for the parameter of interest but one or more of these were inappropriate.	1
10. Summary on the overall risk of study bias	**LOW RISK**	0-3
	**MODERATE RISK**	4-6
	**HIGH RISK**	7-9

For peer review only

Reporting checklist for protocol of a systematic review and meta-analysis.

Based on the PRISMA-P guidelines.

Instructions to authors

Complete this checklist by entering the page numbers from your manuscript where readers will find each of the items listed below.

Your article may not currently address all the items on the checklist. Please modify your text to include the missing information. If you are certain that an item does not apply, please write "n/a" and provide a short explanation.

Upload your completed checklist as an extra file when you submit to a journal.

In your methods section, say that you used the PRISMA-Preporting guidelines, and cite them as:

Moher D, Shamseer L, Clarke M, Ghersi D, Liberati A, Petticrew M, Shekelle P, Stewart LA. Preferred Reporting Items for Systematic Review and Meta-Analysis Protocols (PRISMA-P) 2015 statement. Syst Rev. 2015;4(1):1.

		Reporting Item	Page Number
**Title**			
Identification	#1a	Identify the report as a protocol of a systematic review	1
Update	#1b	If the protocol is for an update of a previous systematic review, identify as such	n/a
**Registration**			
	#2	If registered, provide the name of the registry (such as PROSPERO) and registration number	PROSPERO CRD42021269528
**Authors**			
Contact	#3a	Provide name, institutional affiliation, e-mail address of all protocol authors; provide physical mailing address of corresponding author	1, 2

For peer review only

Contribution	#3b	Describe contributions of protocol authors and identify the guarantor of the review	12
**Amendments**			
	#4	If the protocol represents an amendment of a previously completed or published protocol, identify as such and list changes; otherwise, state plan for documenting important protocol amendments	n/a
**Support**			
Sources	#5a	Indicate sources of financial or other support for the review	13
Sponsor	#5b	Provide name for the review funder and / or sponsor	13
Role of sponsor or funder	#5c	Describe roles of funder(s), sponsor(s), and / or institution(s), if any, in developing the protocol	13
**Introduction**			
Rationale	#6	Describe the rationale for the review in the context of what is already known	5,6
Objectives	#7	Provide an explicit statement of the question(s) the review will address with reference to participants, interventions, comparators, and outcomes (PICO)	6
**Methods**			
Eligibility criteria	#8	Specify the study characteristics (such as PICO, study design, setting, time frame) and report characteristics (such as years considered, language, publication status) to be used as criteria for eligibility for the review	6,7
Information sources	#9	Describe all intended information sources (such as electronic databases, contact with study authors, trial registers or other grey literature sources) with planned dates of coverage	8

For peer review only

Search strategy	#10	Present draft of search strategy to be used for at least one electronic database, including planned limits, such that it could be repeated	9
Study records - data management	#11a	Describe the mechanism(s) that will be used to manage records and data throughout the review	10
Study records - selection process	#11b	State the process that will be used for selecting studies (such as two independent reviewers) through each phase of the review (that is, screening, eligibility and inclusion in meta-analysis)	9,10
Study records - data collection process	#11c	Describe planned method of extracting data from reports (such as piloting forms, done independently, in duplicate), any processes for obtaining and confirming data from investigators	10
Data items	#12	List and define all variables for which data will be sought (such as PICO items, funding sources), any pre-planned data assumptions and simplifications	10
Outcomes and prioritization	#13	List and define all outcomes for which data will be sought, including prioritization of main and additional outcomes, with rationale	7,8
Risk of bias in individual studies	#14	Describe anticipated methods for assessing risk of bias of individual studies, including whether this will be done at the outcome or study level, or both; state how this information will be used in data synthesis	10,11
Data synthesis	#15a	Describe criteria under which study data will be quantitatively synthesised	11
Data synthesis	#15b	If data are appropriate for quantitative synthesis, describe planned summary measures, methods of handling data and methods of combining data from studies, including any planned exploration of consistency (such as I2, Kendall's τ)	11

For peer review only

Data synthesis	#15c	Describe any proposed additional analyses (such as sensitivity or subgroup analyses, meta-regression)	11
Data synthesis	#15d	If quantitative synthesis is not appropriate, describe the type of summary planned	n/a
Meta-bias(es)	#16	Specify any planned assessment of meta-bias(es) (such as publication bias across studies, selective reporting within studies)	10,11
Confidence in cumulative evidence	#17	Describe how the strength of the body of evidence will be assessed (such as GRADE)	12

None The PRISMA-P elaboration and explanation paper is distributed under the terms of the Creative Commons Attribution License CC-BY. This checklist can be completed online using https://www.goodreports.org/, a tool made by the EQUATOR Network in collaboration with Penelope.ai

For peer review only

BMJ Open

Burden of Mental Health Problems Among Pregnant and Post-Partum Women in Sub-Saharan Africa: Systematic Review and Meta-analysis Protocol

Journal:	*BMJ Open*
Manuscript ID	bmjopen-2022-069545.R2
Article Type:	Protocol
Date Submitted by the Author:	17-May-2023
Complete List of Authors:	Awini, Elizabeth; Ghana Health Service<br>Agyepong, Irene; Ghana Health Service; Ghana College of Physicians and Surgeons, Faculty of Public Health<br>Owiredu, David; University of Ghana<br>Gyimah, Leveana; Pantang Hospital; Ghana College of Physicians and Surgeons, Faculty of Psychiatry<br>Ashinyo, Mary; Ghana Health Service, Institutional Care Division<br>Yevoo, Lucy; Ghana Health Service<br>Aye, Sorre Grace Emmanuelle Victoire ; Ghana Health Service, Research and Development Division; Ghana College of Physicians and Surgeons, Faculty of Public Health<br>Abbas, Shazra; The University of Melbourne, Nossal Institute for Global Health, Melbourne School of Population and Global Health<br>Cronin de Chavez, Anna; London School of Hygiene and Tropical Medicine Department of Global Health and Development<br>Kane, Sumit; The University of Melbourne, Melbourne School Of Population And Global Health<br>Mirzoev, Tolib; London School of Hygiene & Tropical Medicine, Global Health and Development<br>Danso-Appiah , Anthony ; University of Ghana, Epidemiology and Disease Control; University of Ghana, Centre for Evidence Synthesis and Policy
**Primary Subject Heading**:	Mental health
Secondary Subject Heading:	Epidemiology, Global health, Public health
Keywords:	MENTAL HEALTH, Maternal medicine < OBSTETRICS, PSYCHIATRY

SCHOLARONE™
Manuscripts

Burden of Mental Health Problems Among Pregnant and Post-Partum Women in Sub-Saharan Africa: Systematic Review and Meta-analysis Protocol

Elizabeth Awini[1], Irene Akua Agyepong[1,2], David Owiredu[3], Leveana Gyimah[4,5], Mary Eyram Ashinyo[6], Linda Lucy Yevoo[1], Sorre Grace Emmanuelle Victoire Aye[1,2], Shazra Abbas[7], Anna Cronin de Chavez[8], Sumit Kane[7], Tolib Mirzoev[8], Anthony Danso-Appiah[3,9,*]

Author affiliation

[1]Dodowa Health Research Centre, Research and Development Division, Ghana Health Service, Dodowa, Ghana
[2]Ghana College of Physicians and Surgeons, Faculty of Public Health, Accra, Ghana
[3]Department of Epidemiology and Disease Control, School of Public Health, University of Ghana, Legon, Ghana
[4]Pantang Hospital, Accra, Ghana
[5]Ghana College of Physicians and Surgeons, Faculty of Psychiatry, Accra, Ghana
[6]Institutional Care Division, Ghana Health Service, Accra, Ghana
[7]Nossal Institute for Global Health, Melbourne School of Population and Global Health, The University of Melbourne, Victoria 3010 Australia
[8]Department of Global Health and Development, London School of Hygiene and Tropical Medicine, UK
[9] Centre for Evidence Synthesis and Policy, University of Ghana, Legon, Accra, Ghana

Author email addresses

Elizabeth Awini; awini.elizabeth@gmail.com
Irene Akua Agyepong; irene.agyepong@gmail.com
David Owiredu; evasupreme@gmail.com
Leveana Gyimah; leveana12@gmail.com
Mary Eyram Ashinyo; mary.ashinyo@ghsmail.org
Linda Lucy Yevoo; lyevoo@gmail.com
Sorre Grace Emmanuelle Victoire Aye; graceaye7@gmail.com
Shazra Abbas; shazra.abbas1@unimelb.edu.au
Anna Cronin de Chavez; Anna.Chavez@lshtm.ac.uk
Sumit Kane; sumit.kane@unimelb.edu.au
Tolib Mirzoev; Tolib.Mirzoev@lshtm.ac.uk

***Corresponding author**

Dr Antony Danso-Appiah, DSc, PhD

1. Department of Epidemiology and Disease Control, School of Public Health
   University of Ghana, P. O. Box LG13, Legon, Accra, Ghana
2. UG Centre for Evidence Synthesis and Policy, School of Public Health
   University of Ghana, P. O. Box LG13, Legon, Accra, Ghana

Email: adanso-appiah@ug.edu.gh
tdappiah@yahoo.co.uk

For peer review only - http://bmjopen.bmj.com/site/about/guidelines.xhtml

Tel: +233 269083789
+44 7958721789

Keywords:
Maternal, mental health, mental disorders, mental conditions, perinatal, pregnant women, postpartum women, burden of disease, Sub-Saharan Africa

ORCID IDs

Elizabeth Awini: **0000-0002-1147-985X**

Irene Akua Agyepong: **0000-0002.0193.5882**

David Owiredu: **0000-0002-4503-1850**

Leveana Gyimah: **0000-0003-2323-4718**

Mary Eyram Ashinyo: **0000-0002-8493-9378**

Linda Lucy Yevoo: **0000-0003-2309-2158**

Aye Sorre Grace Emmanuelle Victoire **0000-0001-7435-677X**

Shazra Abbas **0000-0002-5402-0766**

Anna Cronin de Chavez: **0000-0002-4050-4276**

Sumit Kane: **0000-0002-4858-7344**

Tolib Mirzoev: **0000-0003-2959-9187**

Anthony Danso-Appiah: **0000-0003-1747-0060**

Word count

Main Text Word Count: 2689

Tables: 1

For peer review only

Abstract

Introduction

Pregnancy and postpartum- related mental health problems pose serious public health threat to the society, but worryingly, neglected in Sub-Saharan Africa (SSA). This review will assess the burden and distribution of maternal mental health (MMH) problems in SSA, with the aim to inform the implementation of context sensitive interventions and policies.

Methods and analysis

All relevant databases, grey literature and non-database sources will be searched. PubMed, LILAC, CINAHL, SCOPUS and PsycINFO, Google Scholar, African Index Medicus, HINARI, African Journals Online and IMSEAR will be searched from inception to 31st May 2023, without language restriction. The reference lists of articles will be reviewed, and experts contacted for additional studies missed by our searches. Study selection, data extraction and risk of bias assessment will be done independently by at least two reviewers and any discrepancies will be resolved through discussion between the reviewers. Binary outcomes (prevalence and incidence) of MMH problems will be assessed using pooled proportions, Odds Ratio (OR) or Risk Ratio (RR), and Mean Difference (MD) for continuous outcomes; all will be presented with their 95% confidence intervals (CIs). Heterogeneity will be investigated graphically for overlapping CIs and statistically using the I^2 statistic and where necessary subgroup analyses will be performed. Random-effects model meta-analysis will be conducted when heterogeneity is appreciable, otherwise fixed-effect model will be used. The overall level of evidence will be assessed using GRADE.

Ethics, dissemination and protocol registration

Although no ethical clearance or exemption is needed for a systematic review, this review is part of a larger study on maternal mental health which has received ethical clearance from the Ethics Review Committee of the Ghana Health Service (GHS-ERC 012/03/20). Findings of this study will be disseminated through stakeholder forums, conferences and peer review publications. This systematic review has been registered in PROSPERO- CRD42021269528.

For peer review only - http://bmjopen.bmj.com/site/about/guidelines.xhtml

Strengths and limitations of this study

- This study uses robust methods, best practices and reporting guidelines to attempt to synthesize evidence on the burden of MMH problems in SSA to provide country and regional- specific estimates.
- Screening of articles, data extraction and quality assessment will be done using validated tools by at least two independent reviewers to minimize bias.
- The study uses comprehensive search terms and strategy, and involves relevant electronic databases and non-database sources to attempt to retrieve all potentially relevant studies.
- A possible limitation is, there were no previous data to compare our systematic review to.

Introduction

Maternal mental health (MMH) problems, occurring during pregnancy and postpartum constitute a serious public health problem globally, affecting about 10% of pregnant women and 13% of postpartum mothers[1-3]. A systematic review on global depression among postpartum women estimated prevalence of 17.2% worldwide[4]. In high income countries, prevalence of postpartum depression ranged from 5% to 20%[5]. Data from 2010-2015 Northern Ireland Maternity System indicated that 18.9% of pregnant women reported a history of at least one mental disorder[6]. In the United States, 10% depression in mothers was reported over 12 months[7]. Analysis of Claims data from January to December 2008 involving 38,174 pregnant women in Germany identified at least one mental health problem from four main mental health disorders in 16,639 of the women[8] with somatoform/dissociative disorder being 24.2%, anxiety 16.9%, stress reactions 11.7%, and depression 9.3%. While reliable data are not readily available, existing estimates suggest the burden of MMH problems is relatively higher in low- and middle-income countries (LMICs) with 1 in 4 women reporting depression during pregnancy and 1 in 5 after delivery[9]. A review of evidence from LMICs on pre and postnatal depression reported prevalence of 4.9% to 50%[10] whereas a systematic review involving pre- and postnatal women living in Africa reported prevalence of depression during pregnancy at 11.3% and 18.3 % after delivery[11]. Another review in SSA reported 18.6% post-partum depression[12], with a range from 7% to 50.3%.

The common mental health problems experienced by pregnant and postpartum women are depression and anxiety[13-16]. Depression can be mild, moderate or severe. Mild depression will normally not affect the individual's ability to undertake their day-to-day activities such as self-care and interpersonal relationships whereas moderate to severe episodes can render the mother less capable of undertaking their basic self-care

For peer review only - http://bmjopen.bmj.com/site/about/guidelines.xhtml

and that of their newborn babies which could impact on breastfeeding and bonding[17]. Other maternal mental health disorders are postpartum psychosis, pregnancy and postpartum obsessive-compulsive disorder (OCD), birth-related posttraumatic stress disorder (PTSD), intrusive thoughts and mania, schizophrenia, infanticide, substance use disorder, anorexia nervosa, bulimia, suicidal ideation, bipolar affective disorder, paranoia, psychopathy, neurotic disorders and self-harm[18, 19]. These disorders may develop as a result of experiences associated with childbirth, foetal loss, congenital malformations, intimate partner violence during pregnancy, lack of partner support, history of abuse, unplanned pregnancy, complications in previous pregnancy, higher perceived stress, lower self-esteem[20-24], and many more. Individuals with predisposition to bipolar affective disorders may develop manic episodes from pregnancy-related stress[20]. Severity of maternal mental health disorders can potentially progresses from mild or moderate to severe forms if not identified early and appropriate intervention instituted[25, 26].

Rationale for this systematic review

Mothers with mental health problems may not be able to realize their abilities, work productively or cope with the normal stresses of life[3]. Maternal mental health disorders can have negative effects on both the mother and the child[27]. In severe cases, depressed mothers may have symptoms of a psychosis[4]. Affected mothers often cannot function properly, with some having suicidal tendencies[3]. Evidence shows that maternal mental health problems are associated with negative birth outcomes and may adversely affect cognitive development of the infant[28, 29], nutritional status of their infants[30], and early child wellbeing[31]. There is, however, limited knowledge to inform policy makers on the burden of maternal mental health disorder in SSA to inform on the selection and implementation of locally relevant, feasible, and effective interventions. Maternal mental health is an important health problem which should be given the needed attention but this has often been neglected as a health priority[32-34.] There is much evidence that in LMICs the vast majority of women who experience mental health problems during and after pregnancy do not receive the needed treatment[1, 35]. Although few systematic reviews have investigated mental health problems in SSA, none focused specifically on MMH[36, 37]. The only review that assessed risk factors for antenatal depression, included only studies conducted in Ethiopia[36], and although a systematic review protocol has been registered in PROSPERO[37], it intends to explore the role of MMH disorders and stillbirths.

For peer review only - http://bmjopen.bmj.com/site/about/guidelines.xhtml

This review will assess the burden of MMH problems among pregnant and post-partum women in SSA and attempts to provide robust country and regional estimates of the burden of disease across countries in SSA. Specifically, it will determine the prevalence and incidence and describe the socio-demographic characteristics, general obstetric histories and characteristics of women with MMH problems; assess effects of maternal mental health problems on birth outcomes; and analyze for differences in the burden of mental health problems among pregnant and post-partum women between rural and urban settings. The review will answer the following specific questions: 1) What is the magnitude of MMH problems among pregnant and post-partum women living in SSA? 2) What are the socio-demographic characteristics of pregnant and post-partum women with mental health problems in SSA? 3) What are the general obstetric histories and characteristics of pregnant and post-partum women in SSA with mental health problems? 4) Is there any difference in the burden of pregnant and post-partum women with mental health problems between rural and urban settings? and 6) Are there any documented effects of birth outcomes of pregnant women with mental health problems?

Review methods

This review protocol has been prepared following the Preferred Reporting Items for Systematic Review and Meta-Analysis extension for protocols (PRISMA-P)[38] (Supplemental file 1) and the PRISMA flow diagram (Supplemental file 2). The full review will be prepared in line with the Preferred Reporting Items for Systematic Review and Meta-Analysis (PRISMA). The full review is expected to start on 1st May 2023, analysis 1st September and completed by 30th November 2023.

Patient and public involvement

The review questions and outcome measures have been developed collaboratively with the relevant patient and consumer involvement and informed by their priorities, experience, and preferences in line with GRIPP2 reporting checklists. The review findings will be shared with relevant wider patient communities who will also be involved in the results dissemination.

Criteria for considering studies for this review

Types of studies

Any study (cohort, case-control and cross-sectional studies) conducted in SSA that assessed burden of maternal mental health problems among pregnant and post-partum women will be eligible for inclusion. This review is not an intervention effectiveness review and randomized controlled trials (RCTs)

For peer review only - http://bmjopen.bmj.com/site/about/guidelines.xhtml

are not the focus but if a RCT reported baseline number of cases and used a well-defined sample to serve as the denominator to allow the calculation of proportion/prevalence/incidence in pregnant or postpartum women, such an RCT will be eligible for inclusion. Reviews will not be eligible for inclusion. However, we will go through the reviews to identify potentially eligible studies missed by our searches. If the study is a global review having, for example, SSA or sub-regional sub-set we will extract the studies conducted in SSA for inclusion in this review. If the study reported a country or regional estimate without a well-defined sample (representative sample or sub-sample of the source population), it will not be eligible for inclusion. In cases where the results of a multi-country study have been lumped together and there is no way of disaggregating the data, such studies will not be included. Case studies and case series (these are atypical and not representative of the source population), commentaries or opinions, will not be eligible for inclusion.

Participants

Pregnant and post-partum (postpartum is defined as up to 12 months after delivery) women living in SSA, diagnosed of any mental health disorder (depression, anxiety, postpartum psychosis, bipolar disorders, substance misuse disorders, dysthymia, obsessive-compulsive disorder, posttraumatic stress disorder, schizophrenia, infanticide, suicidal ideation, paranoia, psychopathy, neurosis/neurotic disorders, self-harm, anorexia nervosa, bulimia, etc.) will be eligible for inclusion. The tool or criteria for diagnosis should be stated, for example, standard operational diagnostic criteria such as Research Diagnostic Criteria (RDC)[39], the 10th edition of the International Classification of Diseases (ICD-10)[40] or Diagnostic and Statistical Manual of Mental Disorders (DSM-V)[41]. Pregnant or post-partum women whose diagnosis of mental health disorder could not be confirmed will be excluded.

Interventions

This systematic review is not intervention review.

Comparison

This is non-comparative review but where outcomes or variables permit comparison, we will attempt to compare.

Outcomes

Primary outcomes

- Burden of MMH problems measured as prevalence, incidence etc.
- Birth outcomes (maternal and foetal outcomes) up to 12 months postpartum
- Type of MMH conditions (for diagnosed cases)

For peer review only - http://bmjopen.bmj.com/site/about/guidelines.xhtml

Secondary outcomes

- Proportion of MMH conditions captured in the community
- Predisposition socio-demographic characteristics of pregnant and post-partum women with mental health problems
- Predisposition obstetric histories and characteristics of pregnant and post-partum women in SSA with mental health problems
- Psychiatric predisposition (pre-existing mental health issues triggered during or after pregnancy).
- Burden of mental health problems among pregnant and post-partum women between rural and urban settings

Search strategy

We will search the following electronic databases: PubMed, PsycINFO, LILAC and CINAHL from inception to 31st May 2023 without language restriction and using the search terms in Table 1. We will also search Google Scholar, African Index Medicus, HINARI, African Journals Online, IMSEAR and relevant preprint repositories. Grey literature including dissertations and conference proceedings will be searched. Reference list of retrieved articles will be reviewed, and experts in the field of MMH will be contacted for studies not captured by our searches (see Table 1 for search strategy developed for PubMed).

For peer review only

Table 1 Search Strategy for PubMed (to be adapted for the other databases)

Search	Query	Results
#1	Search: **((((((((((((((((((((((("mental health"[Title/Abstract]) OR ("mental disorder"[Title/Abstract])) OR ("mental illness"[Title/Abstract])) OR ("mental problem"[Title/Abstract])) OR ("chronic mental illness"[Title/Abstract])) OR ("psychiatric illness"[Title/Abstract])) OR ("chronic psychiatric illness"[Title/Abstract])) OR ("chronic insanity"[Title/Abstract])) OR (insanity[Title/Abstract])) OR ("chronic mental disorder"[Title/Abstract])) OR ("dementia praecox"[Title/Abstract])) OR (schizophrenia[Title/Abstract])) OR (psychoses[Title/Abstract])) OR (psychosis[Title/Abstract])) OR ("schizophrenic psychosis"[Title/Abstract])) OR (depression[Title/Abstract])) OR ("mental sickness"[Title/Abstract])) OR ("mental disease"[Title/Abstract])) OR (maladjustment[Title/Abstract])) OR ("emotional disorder"[Title/Abstract])) OR ("nervous disorder"[Title/Abstract])) OR ("nervous breakdown"[Title/Abstract])) OR (neurosis[Title/Abstract])) OR ("neurotic disorder"[Title/Abstract])) OR (psychopathy[Title/Abstract])) OR (paranoia[Title/Abstract])**	
#2	Search: **((((((((((((("pregnant women"[Title/Abstract]) OR (prepartum[Title/Abstract])) OR (peripartum[Title/Abstract])) OR (perinatal[Title/Abstract])) OR (prenatal[Title/Abstract])) OR (antenatal[Title/Abstract])) OR ("during pregnancy"[Title/Abstract])) OR (postpartum[Title/Abstract])) OR (maternal[Title/Abstract])) OR (maternity[Title/Abstract])) OR (parturient[Title/Abstract])) OR (antepartum[Title/Abstract])) OR ("post-delivery"[Title/Abstract])) OR (puerperium[Title/Abstract])**	
#3	Search: **(#1) AND (#2)**	
#4	Search: **((("sub-Saharan Africa") OR (SSA)) OR (Angola)) OR (Benin)) OR (Botswana)) OR ("Burkina Faso")) OR (Burundi)) OR (Cameroon)) OR ("Cape Verde")) OR ("Central African Republic")) OR (Chad)) OR (Comoros)) OR (Congo)) OR ("Cote d'Ivoire")) OR (Djibouti)) OR ("Equatorial Guinea")) OR (Ethiopia)) OR (Gabon)) OR ("The Gambia")) OR (Ghana)) OR (Guinea)) OR ("Guinea-Bissau")) OR (Kenya)) OR (Lesotho)) OR (Liberia)) OR (Madagascar)) OR (Malawi)) OR (Mali)) OR (Mauritania)) OR (Mauritius)) OR (Mozambique)) OR (Namibia)) OR (Niger)) OR (Nigeria)) OR (Rwanda)) OR ("Sao Tome and Principe")) OR (Senegal)) OR (Seychelles)) OR ("Sierra Leone")) OR (Somalia)) OR ("South Africa")) OR (Sudan)) OR (Swaziland)) OR (Tanzania)) OR (Togo)) OR (Uganda)) OR (Zaire)) OR (Zambia)) OR (Zimbabwe)**	
#5	Search: **(#3) AND (#4)**	

The search strategy will be adapted as appropriate for other databases. All searches will be re-run just before the final analyses and any further eligible studies identified will be included.

Study selection

The search output will be managed, collated and de-duplicated using EndNote. The deduplicated articles will be exported to Rayyan[42] for screening and selection. At least two reviewers will screen

For peer review only - http://bmjopen.bmj.com/site/about/guidelines.xhtml

titles and abstracts of identified studies independently using pre-specified and piloted eligibility flowchart (Figure 1). The full text of potentially relevant studies will also be reviewed independently for inclusion. The PRISMA flow diagram will be used to document the flow of studies and reasons for exclusion. Discrepancies will be resolved through discussion between the reviewers.

Data extraction and management

A validated data extraction sheet adapted from the Cochrane Collaboration (Supplemental file 3) will be used by the reviewers to independently extract relevant data. Data to be extracted include characteristics of the studies such as year study was conducted, year study was published (for published studies), country where study was conducted, study design and sample size; socio-demographic characteristics of the participants (age, setting, socio-economic status, level of education and occupation); obstetric factors (parity, maternal age, age at first delivery, history of miscarriage, history of stillbirth), mental health conditions (depression, anxiety, postpartum psychosis, dysthymia/persistent depressive disorder, pregnancy and postpartum obsessive-compulsive disorder (OCD), birth- related posttraumatic stress disorder (PTSD), intrusive thoughts and mania, schizophrenia, infanticide, substance use disorder, anorexia nervosa, bulimia, suicidal ideation, bipolar affective disorder, paranoia, psychopathy, neurotic disorders and self-harm); and quality domains for the assessment of quality of the included studies for risk of bias. Data on burden of the disease (such as prevalence, incidence and duration of the MMH problem) will be extracted. The corresponding authors of the primary studies will be contacted for missing data or unclear information. Where it is not possible to obtain the missing information, data will be analyzed based on those with complete outcome data and the amount of data missing with reasons will be provided. If necessary, data will be coded and recoded before use in the analysis. The extracted data will be verified independently and any disagreement will be resolved through discussion.

Assessment of quality of the included studies

At least two reviewers will assess quality of the included studies for risk of bias (methodology and reporting) independently using the appropriate risk of bias assessment tools. Since this review is not focusing on randomized controlled trials (RCTs), the domains on the Cochrane Risk of Bias (ROB) tool[43] will not be covered fully. Selective outcome and analysis reporting bias (which occurs when studies with positive and significant results are likely to be reported or published) are the domains to be considered on the Cochrane ROB tool. The Cochrane criteria for reporting risk of bias in the included studies will be used to assess pre-specified outcomes of interest. The risk

For peer review only - http://bmjopen.bmj.com/site/about/guidelines.xhtml
For peer review only

of bias will be rated as "low" if protocol of the study is available and all pre-specified outcomes of interest as specified in the protocol, or the protocol is not available but it is clear that all pre-specified and expected outcomes of interest have been reported. The risk of bias will be rated as "high" for a study if outcomes are not reported as pre-specified or expected and "unclear" when there is not enough information to make clear judgement. Other pre-specified biases pertaining to the methods, source of funding etc will be assessed and rated. The Risk of Bias Tool for Prevalence studies will be assessed using the tool by Hoy et al[44] (Supplemental file 4) on four domains: selection bias, non-response bias, measurement bias and bias related to data analysis for prevalence studies. Each risk of bias domain will be graded as 'low risk', 'high risk', and 'unclear' risk of bias. Any disagreements will be resolved through discussion between the reviewers, and if necessary, a third reviewer will be consulted.

Data analysis

Binary outcomes will be assessed using Odds Ratio (OR) or Risk Ratio (RR), and for continuous data, we will use Mean difference (MD) or Standardized Mean Difference (SMD) for means that used different scales. Meta-Analysis, the statistical component of systematic review, will be used to combine study outcomes. Odds Ratio, RR and MD of the individual studies will be pooled and presented with their 95% confidence interval (CI). Random-effects model will be used in the meta-analysis when heterogeneity is high, otherwise fixed-effect model will be used. Descriptive statistics will be used to describe proportions (prevalence and incidence).

Heterogeneity and subgroup analysis

Heterogeneity arises because of variation in the study design, characteristics of participants or outcomes between or within studies[45]. Heterogeneity will be investigated both graphically and statistically. The I^2 statistic which describes the percentage of variability that is due to heterogeneity rather than chance will be estimated. The I^2 is classified into four levels: 0%, 1-29%, 30-59% and 60-100%[46] and I^2>50% indicates significant heterogeneity[47]. Subgroup analysis which is used to estimate an effect of indicator within each subgroup or subset will be used to address heterogeneity due to variation in effects in each subgroup mixed effect model. Thus, the burden of MMH problems will be estimated separated for urban and rural and for each age group. Subgroup analysis will also be done for gestational age and postpartum period. If we find sufficient number of studies, subgroup analysis will also be based on perinatal mental disorder type, study design, parity and location.

For peer review only - http://bmjopen.bmj.com/site/about/guidelines.xhtml

Grading the evidence

The overall evidence of the systematic review will be graded using Grading of Recommendations Assessment, Development and Evaluation (GRADE)[48] (Available from guidelinedevelopment.org/handbook). The GRADE system assesses the following domains: risk of bias, imprecision, inconsistency, indirectness and publication bias, and classifies the quality of evidence as high, moderate, low, and very low. If evidence from a study is graded high quality, it implies further research is very unlikely to change the confidence in the estimate of effect while a grading of very low quality implies an estimate of effect is very doubtful. This protocol has been registered on the International Prospective Register of Systematic Reviews (PROSPERO) with registration **ID CRD42021269528.**

Ethical approval and dissemination

Although no ethical clearance or exemption is needed for a systematic review, this review is part of a larger study on maternal mental health that involves primary data collection and which has received ethical clearance from the ethics review committee of the Ghana Health Service (GHS-ERC 012/03/20)**.** The results of the review will be presented to stakeholders (policymakers and practitioners), It will also be disseminated through conferences and peer review publications.

Author contributions

Protocol conceptualization and design: EA, IAA, SK, TM and ADA

Drafting the work or revising it critically for important intellectual content: EA, IAA, DO, LG, MEA, LLY, SGEVA, SA, ACC, SK, TM and ADA

Acquisition, analysis, or interpretation of data: EA, IAA, DO, LG, MEA, LLY, SGEVA, SA, ACC, SK, TM and ADA

Agreement to be accountable for accuracy or integrity of all aspects of the work: EA, IAA, DO, LG, MEA, LLY, SGEVA, SA, ACC, SK, TM and ADA

Final approval of the version to be published: EA, IAA, DO, LG, MEA, LLY, SGEVA, SA, ACC, SK, TM and ADA

Supervised this work: ADA

Acknowledgments

We thank Dr. Caleb Othieno, University of Nairobi, Kenya, Mrs. Ruth Owusu-Antwi, Komfo Anokye Teaching Hospital, Kwame Nkrumah University of Science and Technology, Kumasi, Ghana and Stephanie Catsaros, Université Paris Cité, France for peer-reviewing this manuscript

For peer review only - http://bmjopen.bmj.com/site/about/guidelines.xhtml

and providing very useful comments. This systematic review was part of capacity building initiative by the UG Centre for Evidence Synthesis and Policy (UGCESP), Africa Communities of Evidence Synthesis and Translation (ACEST) and the RESPONSE Project that are jointly training experts in evidence synthesis and translation across Low and Middle-Income Countries (LMICs).

Funding Statement

This review is part of a wider RESPONSE study, which received funding from the Joint MRC/ESRC/DFID/Wellcome Health Systems Research Initiative (grant ref: MR/T023481/1). The views expressed in this publication are those of the author(s) and not necessarily those of the funders.

Competing of interest: None declared.

For peer review only

For peer review only - http://bmjopen.bmj.com/site/about/guidelines.xhtml

References

1. Kathree T, Selohilwe OM, Bhana A, Petersen I. Perceptions of postnatal depression and health care needs in a South African sample: the “mental” in maternal health care. *BMC Women's Health.* 2014;14(1):1-11.

2. McCauley M, Brown A, Ofosu B, Van Den Broek N. “I just wish it becomes part of routine care”: healthcare providers’ knowledge, attitudes and perceptions of screening for maternal mental health during and after pregnancy: a qualitative study. *BMC psychiatry.* 2019;19(1):1-8.

3. WHO. WHO | Maternal mental health. Available at: https://www.who.int/mental_health/maternal-child/maternal_mental_health/en/. Accessed 20 December 2020, 2020.

4. Wang Z, Liu J, Shuai H, et al. Mapping global prevalence of depression among postpartum women. *Translational psychiatry.* 2021;11(1):1-13.

5. Nilaweera I, Doran F, Fisher J. Prevalence, nature and determinants of postpartum mental health problems among women who have migrated from South Asian to high-income countries: a systematic review of the evidence. *Journal of Affective Disorders.* 2014;166:213-226.

6. Mongan D, Lynch J, Hanna D, et al. Prevalence of self-reported mental disorders in pregnancy and associations with adverse neonatal outcomes: a population-based cross-sectional study. *BMC pregnancy and childbirth.* 2019;19(1):1-10.

7. Ertel KA, Rich-Edwards JW, Koenen KC. Maternal depression in the United States: Nationally representative rates and risks. *Journal of Women's Health.* 2011;20(11):1609-1617.

8. Wallwiener S, Goetz M, Lanfer A, et al. Epidemiology of mental disorders during pregnancy and link to birth outcome: a large-scale retrospective observational database study including 38,000 pregnancies. *Archives of gynecology and obstetrics.* 2019;299(3):755-763.

9. Gelaye B, Rondon MB, Araya R, Williams MA. Epidemiology of maternal depression, risk factors, and child outcomes in low-income and middle-income countries. *The Lancet Psychiatry.* 2016;3(10):973-982.

10. Parsons CE, Young KS, Rochat TJ, Kringelbach ML, Stein A. Postnatal depression and its effects on child development: a review of evidence from low-and middle-income countries. *British medical bulletin.* 2012;101(1):57-79.

11. Sawyer A, Ayers S, Smith H. Pre-and postnatal psychological wellbeing in Africa: a systematic review. *Journal of affective disorders.* 2010;123(1-3):17-29.

12. Woldeyohannes D, Tekalegn Y, Sahiledengle B, Ermias D, Ejajo T, Mwanri L. Effect of postpartum depression on exclusive breast-feeding practices in sub-Saharan Africa countries: a systematic review and meta-analysis. *BMC Pregnancy and Childbirth.* 2021;21(1):1-10.

13. Atif N, Lovell K, Rahman A. Maternal mental health: The missing “m” in the global maternal and child health agenda. Paper presented at: Seminars in perinatology, 2015.

14. Banti S, Mauri M, Oppo A, et al. From the third month of pregnancy to 1 year postpartum. Prevalence, incidence, recurrence, and new onset of depression. Results from the Perinatal Depression–Research & Screening Unit study. *Comprehensive psychiatry.* 2011;52(4):343-351.

15. Garthus-Niegel S, von Soest T, Knoph C, Simonsen TB, Torgersen L, Eberhard-Gran M. The influence of women’s preferences and actual mode of delivery on post-traumatic stress symptoms following childbirth: a population-based, longitudinal study. *BMC pregnancy and childbirth.* 2014;14(1):1-10.

16. Falah-Hassani K, Shiri R, Dennis C-L. Prevalence and risk factors for comorbid postpartum depressive symptomatology and anxiety. *Journal of affective disorders.* 2016;198:142-147.

For peer review only - http://bmjopen.bmj.com/site/about/guidelines.xhtml

17. Park LT, Zarate Jr CA. Depression in the primary care setting. *New England Journal of Medicine.* 2019;380(6):559-568.

18. Bauer A, Parsonage M, Knapp M, Iemmi V, Adelaja B, Hogg S. The costs of perinatal mental health problems. *London: Centre for Mental Health and London School of Economics.* 2014.

19. Collardeau F, Corbyn B, Abramowitz J, Janssen PA, Woody S, Fairbrother N. Maternal unwanted and intrusive thoughts of infant-related harm, obsessive-compulsive disorder and depression in the perinatal period: study protocol. *BMC psychiatry.* 2019;19(1):1-15.

20. Biaggi A, Conroy S, Pawlby S, Pariante CM. Identifying the women at risk of antenatal anxiety and depression: a systematic review. *Journal of affective disorders.* 2016;191:62-77.

21. Silva MMdJ, Nogueira DA, Clapis MJ, Leite EPRC. Anxiety in pregnancy: prevalence and associated factors. *Revista da Escola de Enfermagem da USP.* 2017;51.

22. Ogbo FA, Eastwood J, Hendry A, et al. Determinants of antenatal depression and postnatal depression in Australia. *BMC psychiatry.* 2018;18(1):1-11.

23. Milgrom J, Hirshler Y, Reece J, Holt C, Gemmill AW. Social support—a protective factor for depressed perinatal women? *International journal of environmental research and public health.* 2019;16(8):1426.

24. Koh M, Ahn S, Kim J, Park S, Oh J. Pregnant women's antenatal depression and influencing factors. *Korean Journal of Women Health Nursing.* 2019;25(1):112-123.

25. Sadock BSV. Postpartum Psychiatric Syndromes. *Comprehensive Textbook of Psychiatry.* Vol 1. 7 ed: Lippincott & Wilkins Publishers.; 2000:992–1053.

26. Semple D, & Smyth, R. Disorders related to childbirth. *Oxford Handbook of Psychiatry*. 3rd ed. Oxford: Oxford University Press; 2013:470–472.

27. O'hara MW, McCabe JE. Postpartum depression: current status and future directions. *Annu Rev Clin Psychol.* 2013;9(1):379-407.

28. Tran TD, Biggs B-A, Tran T, et al. Impact on infants' cognitive development of antenatal exposure to iron deficiency disorder and common mental disorders. *Plos one.* 2013;8(9):e74876.

29. Paschetta E, Berrisford G, Coccia F, et al. Perinatal psychiatric disorders: an overview. *American Journal of Obstetrics and Gynecology.* 2014;210(6):501-509. e506.

30. Hassan BK, Werneck GL, Hasselmann MH. Maternal mental health and nutritional status of six-month-old infants. *Revista de saude publica.* 2016;50:7.

31. MacGinty RP, Lesosky M, Barnett W, Stein DJ, Zar HJ. Associations between maternal mental health and early child wheezing in a South African birth cohort. *Pediatric pulmonology.* 2018;53(6):741-754.

32. O'Mahony JM, Donnelly TT. How does gender influence immigrant and refugee women's postpartum depression help-seeking experiences? *Journal of psychiatric and mental health nursing.* 2013;20(8):714-725.

33. Bagadia A, Chandra PS. Starting the conversation-Integrating mental health into maternal health care in India. *The Indian journal of medical research.* 2017;145(3):267.

34. Cheung FK, Snowden LR. Community mental health and ethnic minority populations. *Community Mental Health Journal.* 1990;26(3):277-291.

35. Fonseca A, Canavarro MC. Women's intentions of informal and formal help-seeking for mental health problems during the perinatal period: The role of perceived encouragement from the partner. *Midwifery.* 2017;50:78-85.

36. Getinet W, Amare T, Boru B, Shumet S, Worku W, Azale T. Prevalence and risk factors for antenatal depression in Ethiopia: systematic review. *Depression research and treatment.* 2018;2018.

For peer review only - http://bmjopen.bmj.com/site/about/guidelines.xhtml

37. Adane AA, Bailey HD, Marriott R, et al. Role of maternal mental health disorders on stillbirth and infant mortality risk: a protocol for a systematic review and meta-analysis. *BMJ open.* 2020;10(5):e036280.

38. Moher D, Shamseer L, Clarke M, et al. Preferred reporting items for systematic review and meta-analysis protocols (PRISMA-P) 2015 statement. *Systematic reviews.* 2015;4(1):1-9.

39. Spitzer RL EJ, Robins E. Research Diagnostic Criteria: Rationale and Reliability. *Arch Gen Psychiatry.* 1978;35(6):773–782.

40. WHO. The ICD-10 Classification of Mental and Behavioural Disorders. *Clinical description and diagnostic guidelines*; 1993.

41. Nuckols CC, Nuckols CC. The diagnostic and statistical manual of mental disorders, (DSM-5). *Philadelphia: American Psychiatric Association.* 2013.

42. Harrison H, Griffin SJ, Kuhn I, Usher-Smith JA. Software tools to support title and abstract screening for systematic reviews in healthcare: an evaluation. *BMC medical research methodology.* 2020;20(1):1-12.

43. Higgins JP, Altman DG, Gøtzsche PC, et al. The Cochrane Collaboration's tool for assessing risk of bias in randomised trials. *Bmj.* 2011;343.

44. Hoy D, Brooks P, Woolf A, et al. Assessing risk of bias in prevalence studies: modification of an existing tool and evidence of interrater agreement. *Journal of clinical epidemiology.* 2012;65(9):934-939.

45. Spineli LM, Pandis N. Exploring heterogeneity in meta-analysis: Subgroup analysis. Part 1. *American journal of orthodontics and dentofacial orthopedics.* 2020;158(2):302-304. e301.

46. Koletsi D, Fleming PS, Michelaki I, Pandis N. Heterogeneity in Cochrane and non-Cochrane meta-analyses in orthodontics. *Journal of dentistry.* 2018;74:90-94.

47. Chang TH, Chou CC, Chang LY. Effect of obesity and body mass index on coronavirus disease 2019 severity: a systematic review and meta-analysis. *Obesity Reviews.* 2020;21(11):e13089.

48. Schünemann H. *The GRADE handbook*: Cochrane Collaboration; 2013.

For peer review only - http://bmjopen.bmj.com/site/about/guidelines.xhtml

Figure Study selection flowchart

For peer review only

For peer review only - http://bmjopen.bmj.com/site/about/guidelines.xhtml

Figure 1 Study selection flowchart

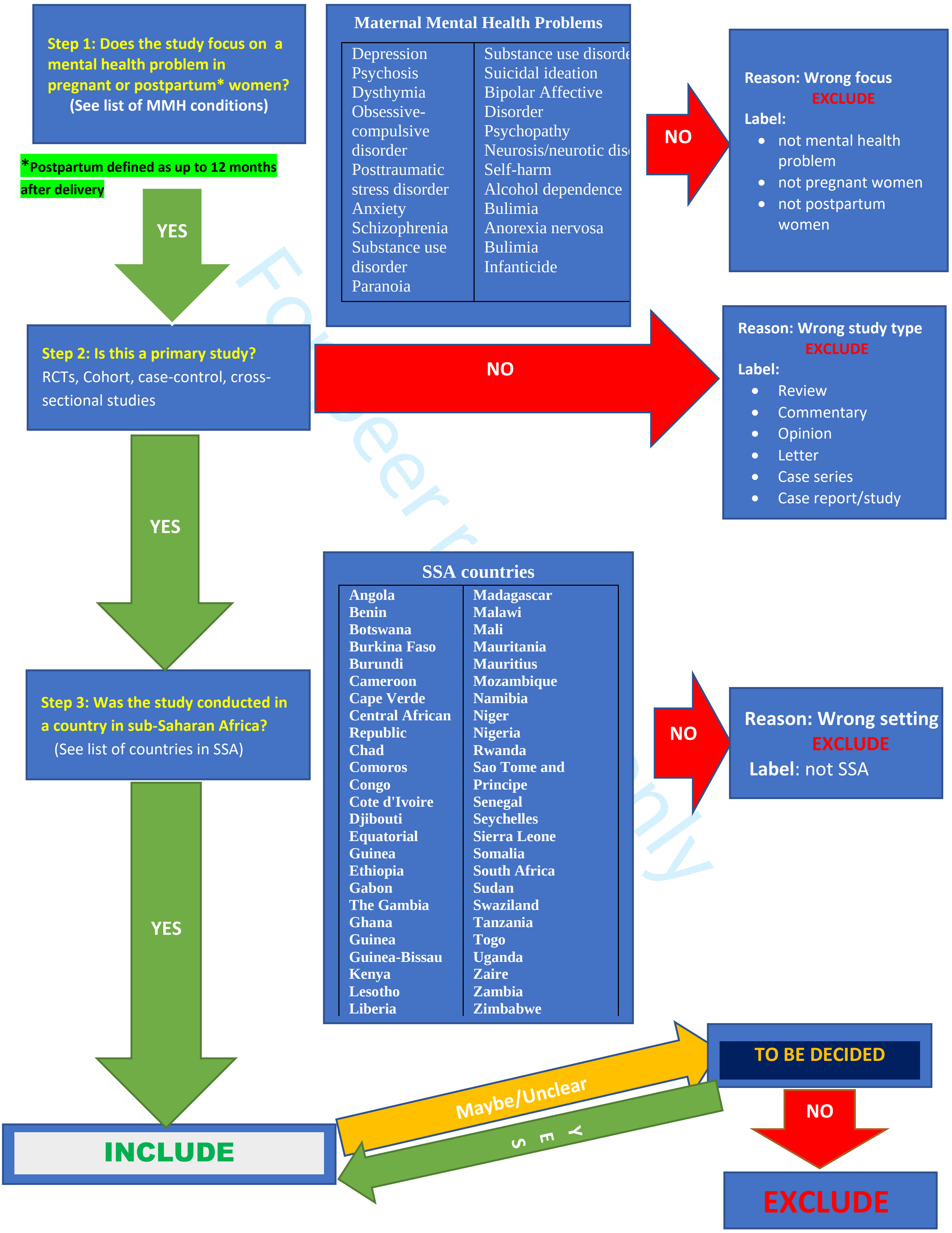

For peer review only

Supplemental file 1 Preferred Reporting Items for Systematic review and Meta-Analysis Protocols (PRISMA-P) checklist*

Section and topic	Item No	Checklist item
**ADMINISTRATIVE INFORMATION**		
Title:		
Identification	1a	Identify the report as a protocol of a systematic review
Update	1b	If the protocol is for an update of a previous systematic review, identify as such
Registration	2	If registered, provide the name of the registry (such as PROSPERO) and registration number
Authors:		
Contact	3a	Provide name, institutional affiliation, e-mail address of all protocol authors; provide physical mailing address of corresponding author
Contributions	3b	Describe contributions of protocol authors and identify the guarantor of the review
Amendments	4	If the protocol represents an amendment of a previously completed or published protocol, identify as such and list changes; otherwise, state plan for documenting important protocol amendments
Support:		
Sources	5a	Indicate sources of financial or other support for the review
Sponsor	5b	Provide name for the review funder and/or sponsor
Role of sponsor or funder	5c	Describe roles of funder(s), sponsor(s), and/or institution(s), if any, in developing the protocol
**INTRODUCTION**		
Rationale	6	Describe the rationale for the review in the context of what is already known
Objectives	7	Provide an explicit statement of the question(s) the review will address with reference to participants, interventions, comparators, and outcomes (PICO)
**METHODS**		
Eligibility criteria	8	Specify the study characteristics (such as PICO, study design, setting, time frame) and report characteristics (such as years considered, language, publication status) to be used as criteria for eligibility for the review
Information sources	9	Describe all intended information sources (such as electronic databases, contact with study authors, trial registers or other grey literature sources) with planned dates of coverage
Search strategy	10	Present draft of search strategy to be used for at least one electronic database, including planned limits, such that it could be repeated
Study records:		
Data management	11a	Describe the mechanism(s) that will be used to manage records and data throughout the review
Selection process	11b	State the process that will be used for selecting studies (such as two independent reviewers) through each phase of the review (that is, screening, eligibility and inclusion in meta-analysis)
Data collection process	11c	Describe planned method of extracting data from reports (such as piloting forms, done independently, in duplicate), any processes for obtaining and confirming data from investigators
Data items	12	List and define all variables for which data will be sought (such as PICO items, funding sources), any pre-planned data assumptions and simplifications
Outcomes and prioritization	13	List and define all outcomes for which data will be sought, including prioritization of main and additional outcomes, with rationale

For peer review only

Risk of bias in individual studies	14	Describe anticipated methods for assessing risk of bias of individual studies, including whether this will be done at the outcome or study level, or both; state how this information will be used in data synthesis
Data synthesis	15a	Describe criteria under which study data will be quantitatively synthesized
	15b	If data are appropriate for quantitative synthesis, describe planned summary measures, methods of handling data and methods of combining data from studies, including any planned exploration of consistency (such as I^2, Kendall's τ)
	15c	Describe any proposed additional analyses (such as sensitivity or subgroup analyses, meta-regression)
	15d	If quantitative synthesis is not appropriate, describe the type of summary planned
Meta-bias(es)	16	Specify any planned assessment of meta-bias(es) (such as publication bias across studies, selective reporting within studies)
Confidence in cumulative evidence	17	Describe how the strength of the body of evidence will be assessed (such as GRADE)

* It is strongly recommended that this checklist be read in conjunction with the PRISMA-P Explanation and Elaboration (cite when available) for important clarification on the items. Amendments to a review protocol should be tracked and dated. The copyright for PRISMA-P (including checklist) is held by the PRISMA-P Group and is distributed under a Creative Commons Attribution Licence 4.0.

For peer review only

Supplemental file 2 PRISMA flow diagram

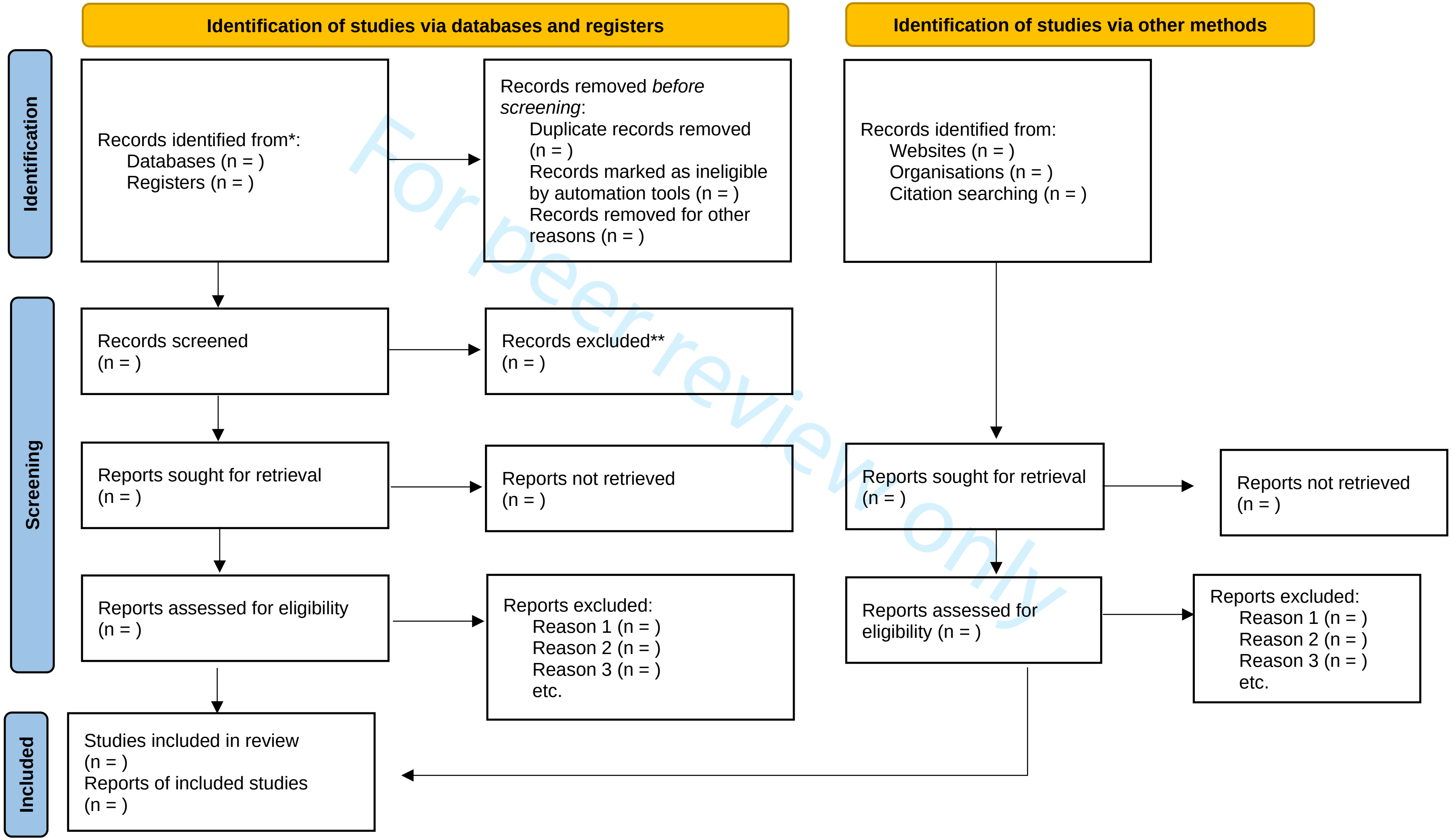

For peer review only

Supplemental file 3 Data extraction sheet (adapted from Cochrane)

**1. General Information**		
1. **Date form completed** *(dd/mm/yyyy)*		
2. **Name/ID of person extracting data**		
3. **Report title** *(title of paper/ abstract/ report that data are extracted from)*		
4. **Publication type** *(e.g. full report, abstract, letter)*		
5. **Study ID** *(e.g. 01 plus surname of first author and year first full report of study was published e.g. Adjuik 2018)*		
6. **Country in which the study conducted**		
7. **Study funding source** *(including role of funders)*		
8. **Possible conflicts of interest** *(for study authors e.g. not reported)*		
**9. Study Characteristics**	**Review Inclusion Criteria** *(as defined in the Protocol)*	**Location in Text** *(page#)*
10. **Type of study**		
11. **Population description**		
12. **Focused diseases / conditions**	*Maternal mental disorders*	
13. **Types of outcome measures**	*Prevalence/incidence/risk ratios, odds ratios/mean difference/proportions*	
14. **Population description** *(from which study participants are drawn)*		
15.. **Source/setting of the population** *(e.g. urban, rural)*		
16. **Method/s of recruitment of participants**		
17. **Aim of study**		
18. **Design** *(e.g. cross-sectional study, cohort study, case-control study etc)*		
19. **Sampling technique** *(e.g. random or convenience)*		
20. **Study start date**		

21. **Study End date/duration** *(if any cohort)*		
22. **Notes:**		
23. **Total number of participants/Sample size**		
24. **Age group**		
25. **Parity**		
26. **Status of mother**	Pregnant, Post-partum	
27. **Outcomes** *(physical/observation examination: who examined?)*		
28. **Self-reported reported outcomes** *(detected by questionnaire/Tools: validated or non-validated?)*		
29. **Outcome names** *(depression, anxiety, psychosis, Birth Related Posttraumatic Stress Disorder, etc)*		
30. **Time points measured** *(report the start month/year/specify whether from start and end of intervention)*		
31. **Time points reported**		
37. **Outcome definition** *(e.g. whether standard case definition used)*		
32. **Type of measurement (***Percentage/Odds ratio/Risk ratio)*		
33. **Is outcome/tool validated?** *(Yes/No/Unclear/Not mentioned)*		
34. **Subgroup** *(if any, e.g. age-specific prevalence reporting)*		
35. **Results**		
36. **Response/non-response rate**		
37. **Any other results reported**		
38. **Unit of analysis** *(e.g. by individuals)*		
39. **Statistical methods used and appropriateness of these methods** *(e.g. proportion/%s, RR/OR)*		

For peer review only

40. **Whether results weighted?** *(e.g. Yes/No)*		
41. **Any other results reported**		
42. **Unit of analysis** *(e.g. by individuals)*		
43. **Statistical methods used and appropriateness of these methods** *(e.g. proportion/%s, RR/OR)*		
44. **All systematic and random error adjusted?** *(e.g. confounding, effect medication etc.)*		
45. **Strength of study**		
46. **Limitation**		
47. **Strategies to overcome the limitation**		
48. **Key conclusions of study authors**		
49. **Notes:**		

For peer review only

Supplemental file 4 Quality assessment checklist for prevalence studies (adapted from Hoy et al., 2012)

Name of author(s):

Year of publication:

Study title:

Risk of bias items	Risk of bias levels	Points scored
1.Was the study's target population a close representation of the national population in relation to relevant variables, e.g. age, sex, occupation?	**Yes (LOW RISK)**: The study's target population was a close representation of the national population.	0
	**No (HIGH RISK)**: The study's target population was clearly NOT representative of the national population.	1
2. Was the sampling frame a true or close representation of the target population?	**Yes (LOW RISK)**: The sampling frame was a true or close representation of the target population.	0
	**No (HIGH RISK)**: The sampling frame was NOT a true or close representation of the target population.	1
3.Was some form of random selection used to select the sample, OR, was a census undertaken?	**Yes (LOW RISK)**: A census was undertaken, OR, some form of random selection was used to select the sample (e.g. simple random sampling, stratified random sampling, cluster sampling, systematic sampling).	0
	**No (HIGH RISK)**: A census was NOT undertaken, AND some form of random selection was NOT used to select the sample.	1
4. Was the likelihood of non-response bias minimal?	**Yes (LOW RISK)**: The response rate for the study was ≥75%, OR, an analysis was performed that showed no significant difference in relevant demographic characteristics between responders and non- responders	0
	**No (HIGH RISK)**: The response rate was <75%, and if any analysis comparing responders and non-responders was done, it showed a significant difference in relevant demographic characteristics between responders and non-responders	1
5. Were data collected directly from the subjects (as opposed to a proxy)?	**Yes (LOW RISK)**: All data were collected directly from the subjects.	0
	**No (HIGH RISK)**: In some instances, data were collected from a proxy.	1

For peer review only

6.Was an acceptable case definition used in the study?	**Yes (LOW RISK)**: An acceptable case definition was used.	0
	**No (HIGH RISK)**: An acceptable case definition was NOT used	1
7. Was the study instrument that measured the parameter of interest (e.g. prevalence of low back pain) shown to have reliability and validity (if necessary)?	**Yes (LOW RISK)**: The study instrument had been shown to have reliability and validity (if this was necessary), e.g. test-re- test, piloting, validation in a previous study, etc.	0
	**No (HIGH RISK)**: The study instrument had NOT been shown to have reliability or validity (if this was necessary).	1
8. Was the same mode of data collection used for all subjects?	**Yes (LOW RISK)**: The same mode of data collection was used for all subjects.	0
	**No (HIGH RISK)**: The same mode of data collection was NOT used for all subjects.	1
9.Were the numerator(s) and denominator(s) for the parameter of interest appropriate	**Yes (LOW RISK)**: The paper presented appropriate numerator(s) AND denominator(s) for the parameter of interest (e.g. the prevalence of low back pain).	0
	**No (HIGH RISK)**: The paper did present numerator(s) AND denominator(s) for the parameter of interest but one or more of these were inappropriate.	1
10. Summary on the overall risk of study bias	**LOW RISK**	0-3
	**MODERATE RISK**	4-6
	**HIGH RISK**	7-9

For peer review only

Reporting checklist for protocol of a systematic review and meta-analysis.

Based on the PRISMA-P guidelines.

Instructions to authors

Complete this checklist by entering the page numbers from your manuscript where readers will find each of the items listed below.

Your article may not currently address all the items on the checklist. Please modify your text to include the missing information. If you are certain that an item does not apply, please write "n/a" and provide a short explanation.

Upload your completed checklist as an extra file when you submit to a journal.

In your methods section, say that you used the PRISMA-Preporting guidelines, and cite them as:

Moher D, Shamseer L, Clarke M, Ghersi D, Liberati A, Petticrew M, Shekelle P, Stewart LA. Preferred Reporting Items for Systematic Review and Meta-Analysis Protocols (PRISMA-P) 2015 statement. Syst Rev. 2015;4(1):1.

		Reporting Item	Page Number
**Title**			
Identification	#1a	Identify the report as a protocol of a systematic review	1
Update	#1b	If the protocol is for an update of a previous systematic review, identify as such	n/a
**Registration**			
	#2	If registered, provide the name of the registry (such as PROSPERO) and registration number	PROSPERO CRD42021269528
**Authors**			
Contact	#3a	Provide name, institutional affiliation, e-mail address of all protocol authors; provide physical mailing address of corresponding author	1, 2

For peer review only

Contribution	#3b	Describe contributions of protocol authors and identify the guarantor of the review	12
**Amendments**			
	#4	If the protocol represents an amendment of a previously completed or published protocol, identify as such and list changes; otherwise, state plan for documenting important protocol amendments	n/a
**Support**			
Sources	#5a	Indicate sources of financial or other support for the review	13
Sponsor	#5b	Provide name for the review funder and / or sponsor	13
Role of sponsor or funder	#5c	Describe roles of funder(s), sponsor(s), and / or institution(s), if any, in developing the protocol	13
**Introduction**			
Rationale	#6	Describe the rationale for the review in the context of what is already known	5,6
Objectives	#7	Provide an explicit statement of the question(s) the review will address with reference to participants, interventions, comparators, and outcomes (PICO)	6
**Methods**			
Eligibility criteria	#8	Specify the study characteristics (such as PICO, study design, setting, time frame) and report characteristics (such as years considered, language, publication status) to be used as criteria for eligibility for the review	6,7
Information sources	#9	Describe all intended information sources (such as electronic databases, contact with study authors, trial registers or other grey literature sources) with planned dates of coverage	8

For peer review only

Search strategy	#10	Present draft of search strategy to be used for at least one electronic database, including planned limits, such that it could be repeated	9
Study records - data management	#11a	Describe the mechanism(s) that will be used to manage records and data throughout the review	10
Study records - selection process	#11b	State the process that will be used for selecting studies (such as two independent reviewers) through each phase of the review (that is, screening, eligibility and inclusion in meta-analysis)	9,10
Study records - data collection process	#11c	Describe planned method of extracting data from reports (such as piloting forms, done independently, in duplicate), any processes for obtaining and confirming data from investigators	10
Data items	#12	List and define all variables for which data will be sought (such as PICO items, funding sources), any pre-planned data assumptions and simplifications	10
Outcomes and prioritization	#13	List and define all outcomes for which data will be sought, including prioritization of main and additional outcomes, with rationale	7,8
Risk of bias in individual studies	#14	Describe anticipated methods for assessing risk of bias of individual studies, including whether this will be done at the outcome or study level, or both; state how this information will be used in data synthesis	10,11
Data synthesis	#15a	Describe criteria under which study data will be quantitatively synthesised	11
Data synthesis	#15b	If data are appropriate for quantitative synthesis, describe planned summary measures, methods of handling data and methods of combining data from studies, including any planned exploration of consistency (such as I2, Kendall's τ)	11

For peer review only

Data synthesis	#15c	Describe any proposed additional analyses (such as sensitivity or subgroup analyses, meta-regression)	11
Data synthesis	#15d	If quantitative synthesis is not appropriate, describe the type of summary planned	n/a
Meta-bias(es)	#16	Specify any planned assessment of meta-bias(es) (such as publication bias across studies, selective reporting within studies)	10,11
Confidence in cumulative evidence	#17	Describe how the strength of the body of evidence will be assessed (such as GRADE)	12

None The PRISMA-P elaboration and explanation paper is distributed under the terms of the Creative Commons Attribution License CC-BY. This checklist can be completed online using https://www.goodreports.org/, a tool made by the EQUATOR Network in collaboration with Penelope.ai

For peer review only
